# Discovery of Anti-inflammatory Ingredients in Chinese Herbal Formula Kouyanqing Granule based on Relevance Analysis between Chemical Characters and Biological Effects

**DOI:** 10.1038/srep18080

**Published:** 2015-12-10

**Authors:** Hong Liu, Yan-fang Zheng, Chu-yuan Li, Yu-ying Zheng, De-qin Wang, Zhong Wu, Lin Huang, Yong-gang Wang, Pei-bo Li, Wei Peng, Wei-wei Su

**Affiliations:** 1 Guangzhou Quality R&D Center of Traditional Chinese Medicine, Guangdong Key Laboratory of Plant Resources, School of Life Sciences, Sun Yat-sen University, Guangzhou, P.R. China; 2 Hutchison Whampoa Guangzhou Baiyunshan Chinese Medicine Co., Ltd., Guangzhou, P.R. China

## Abstract

Kouyanqing Granule (KYQG) is a traditional Chinese herbal formula composed of *Flos lonicerae* (FL), *Radix scrophulariae* (RS), *Radix ophiopogonis* (RO), *Radix asparagi* (RA), and *Radix et rhizoma glycyrrhizae* (RG). In contrast with the typical method of separating and then biologicalily testing the components individually, this study was designed to establish an approach in order to define the core bioactive ingredients of the anti-inflammatory effects of KYQG based on the relevance analysis between chemical characters and biological effects. Eleven KYQG samples with different ingredients were prepared by changing the ratios of the 5 herbs. Thirty-eight ingredients in KYQG were identified using Ultra-fast liquid chromatography-Diode array detector-Quadrupole-Time-of-flight-Tandem mass spectrometry (UFLC-DAD-Q-TOF-MS/MS) technology. Human oral keratinocytes (HOK) were cultured for 24 hours with 5% of Cigarette smoke extract (CSE) to induce inflammation stress. Interleukin-1β (IL-1β), interleukin-6 (IL-6), interleukin-8 (IL-8), and tumour necrosis factor-α (TNF-α) were evaluated after treatment with the eleven KYQG samples. Grey relational analysis(GRA), Pearson’s correlations (PCC), and partial least-squares (PLS) were utilized to evaluate the contribution of each ingredient. The results indicated that KYQG significantly reduced interleukin-1β, interleukin-6, interleukin-8, and tumour necrosis factor-α levels, in which lysine, γ-aminobutyric acid, chelidonic acid, tyrosine, harpagide, neochlorogenic acid, chlorogenic acid, cryptochlorogenic acid, isoquercitrin, luteolin-7-o-glucoside, 3,4-dicaffeoylquinic acid, 3,5-dicaffeoylquinic acid, angoroside C, harpagoside, cinnamic acid, and ruscogenin play a vital role.

Traditional Chinese medicine (TCM) has made eminent contributions to human health in East Asia over the past thousand years, owning the advantages that their effectiveness testified directly on numerous individuals initially in ancient times. Recently, TCM has drawn increasing interest worldwide for its complementary and alternative therapies compared with Western medicine and its remarkable ability to address various difficult diseases^1^,^2^,^3^. Herbal formulas are developed from combinations of various herbs at appropriate doses guided by TCM theory. These formulas contain multiple ingredients and affect numerous targets^4^. Bioactive ingredients in herbal formulas are responsible for the therapeutic effects and are essential to improve quality standards^5^,^6^. However, the bioactive ingredients in most herbal formulas are unclear. Current quality control methods for herbal formulas in China rather than bioactivities are merely based on physical and chemical analyses^7^. To develop herbal formulas, it is urgently necessary to define and perform proper controls of the core bioactive ingredients.

The association between the TCM chemical characteristics and bioactive effects was first proposed in 2002^8^. Over the past decade, more and more meaningful trials aiming to connect TCM ingredients and bioactive effects have been published^9^,^10^,^11^. For example, a researcher in China discovered the core bioactive components effective against blood circulation disorders in a traditional Chinese herbal formula Compound Xueshuantong Capsule by analysing the association between the data of high-performance liquid chromatography (HPLC) characteristic peaks and bioactive effects^9^. These researches focused more on the bioactive ingredients than on single or multiple components with relatively high contents in herbs, and such explorations have provided valuable references for quality control, herb classification, TCM prescription optimization, new medicinal plant identification, and drug discovery^12^,^13^.

Kouyanqing Granule (KYQG) is a traditional Chinese herbal formula composed of *Flos lonicerae* (FL, flower bud of *Lonicera macranthoides* Hand.-Mazz.), *Radix scrophulariae* (RS, root of *Scrophularia ningpoensis* Hemsl.), *Radix ophiopogonis* (RO, root of *Ophiopogon japonicus* (Thunb.) Ker-Gawl.), *Radix asparagi* (RA, root of *Asparagus cochinchinensis* (Lour.) Merr.), and *Radix et rhizoma glycyrrhizae* (RG, root of *Glycyrrhiza uralensis* Fisch.). KYQG is well known in China for its curative effects against inflammatory disorders of the mouth and throat, such as recurrent aphthous ulcers, traumatic ulcers, oral leukoplakia, and oral lichen planus^14^,^15^. The bioactive ingredients in KYQG that exert anti-inflammatory effects remain unknown. In contrast with the typical method of separating and then biologically testing the components individually, this study was designed to establish an approach using a relevance analysis between the chemical characters from UFLC-DAD-Q-TOF-MS/MS (Ultra-fast liquid chromatography-Diode array detector-Quadrupole-Time-of-flight-Tandem mass spectrometry) technology and *in vitro* anti-inflammatory effects, ultimately defining the core bioactive ingredients in KYQG.

## Results

### Preparation of KYQG samples

Uniform design (UD), a method particularly suitable for multi-factor, multi-level experiments, was proposed by Fang and Wang based on the Quasi-Monte Carlo method or number-theoretic method^16^. UD has the significant merit of producing samples with high representativeness in the experimental domain. A five-factor, eleven-level UD was adopted to insure differences among the eleven KYQG samples (Table 1).

### UFLC-DAD-Q-TOF-MS/MS analysis

**Figure 1:**
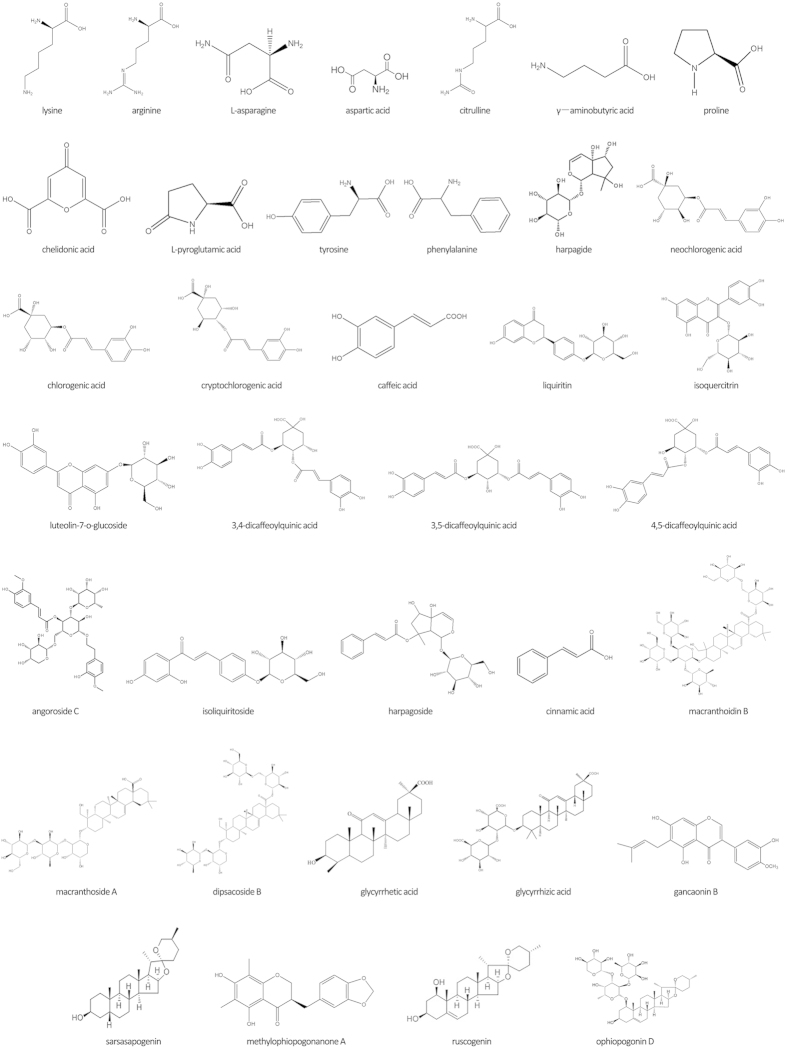
Chemical structural formula of the identified ingredients in KYQG (except for the conjectural saponin 1 and saponin 2^23^).

**Figure 2:**
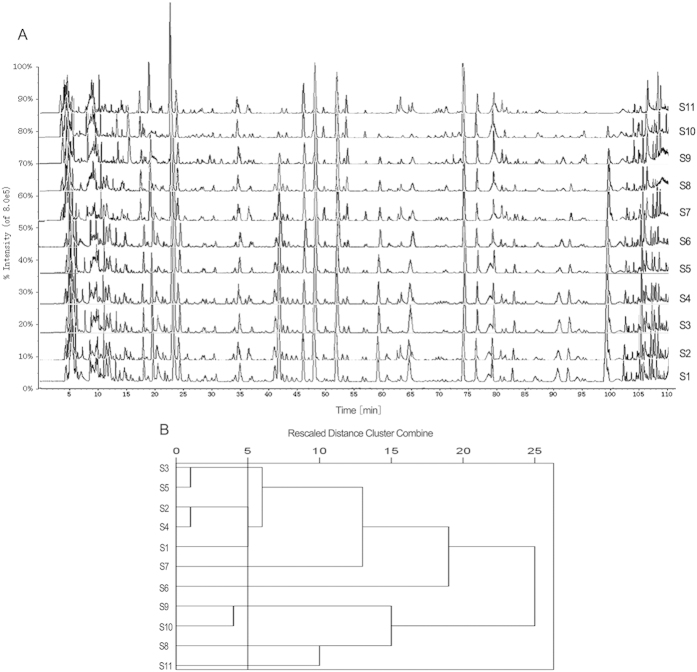
UFLC-Q-TOF-MS/MS characters (**A**) and cluster analysis results (**B**) of eleven KYQG samples. Twenty-five ingredients were confirmed by the retention time. The MS data of the reference substances and thirteen ingredients were characterized by MS data and related studies. Given the rescaled distance of 5, eleven KYQG samples could be divided into eight classes as follows: S2 and S4 belonged to one class, S3 and S5 belonged to one class, S9 and S10 belonged to one class, and the remainder of the samples constituted their own class.

Thirty-eight ingredients were identified or tentatively characterized via UFLC-DAD-Q-TOF-MS/MS. Among the thirty-eight ingredients, twenty-five ingredients were confirmed by retention time and MS data of reference substances, and thirteen ingredients were characterized by MS data and related studies^17^,^18^,^19^,^20^,^21^,^22^,^23^,^24^,^25^ (Table 2, Fig. 1). Given the rescaled distance was 5, cluster analysis results revealed that eleven KYQG samples could be divided into eight classes as follows: S2 and S4 belonged to one class, S3 and S5 belonged to one class, S9 and S10 belonged to one class, and other samples comprised their own class (Fig. 2).

### Evaluation of *anti-inflammatory* activity *in vitro*

#### Cell viability assay

**Figure 3:**
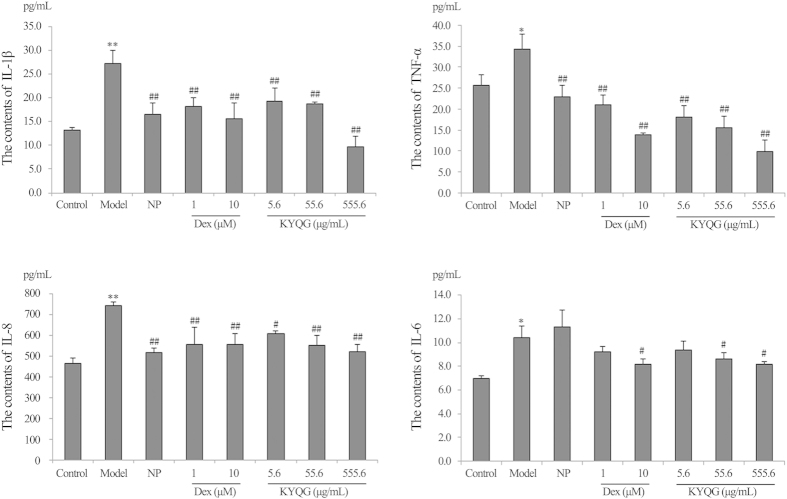
The effects of KYQG (the original formula) on IL-1β, TNF-α, IL-8, and IL-6 production. Dex: Dexamethasone; NP: Niuhuangjiedu Pian; KYQG: Kouyanqing Granule. Groups: control group, model group, NP group (10 μg/mL), Dex groups (1 and 10 μM), and KYQG groups (5.6, 55.6, and 555.6 μg/mL). The control group and model group received the same volume of RPMI-1640 medium for the treatment. Each bar represents the contents of IL-1β, TNF-α, IL-8, and IL-6 as the mean ± SD, n = 3. ^*^P < 0.05 and ^**^P < 0.01 vs. control group, ^#^*P* < 0.05 and ^##^*P* < 0.01 vs. model group.

**Figure 4:**
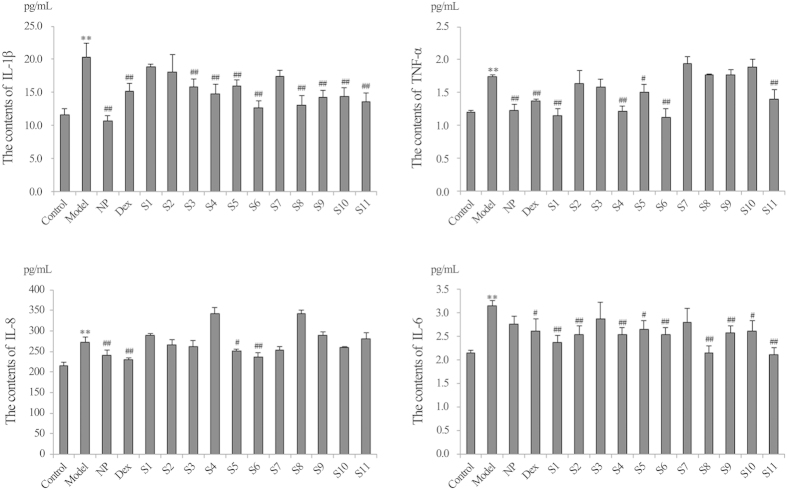
The effects of eleven KYQG samples on IL-1β, TNF-α, IL-8, and IL-6 production. Dex: Dexamethasone; NP: Niuhuangjiedu Pian; KYQG: Kouyanqing Granule. Groups: control group, model group, NP group (10 μg/mL), Dex group (1 μM), and eleven KYQG samples groups (55.6 μg/mL). Control group and model group received the same volume of RPMI-1640 medium for the treatment. Each bar represents the contents of IL-1β, TNF-α, IL-8, and IL-6 as the mean ± SD, n = 3. ^*^P < 0.05 and ^**^P < 0.01 vs. control group, ^#^*P* < 0.05 and ^##^*P* < 0.01 vs. model group.

Cigarette smoke extract (CSE, 1-5%), dexamethasone (Dex, 1–100 μM), Niuhuangjiedu Pian (NP, 10-100 μg/mL), KYQG of original formula (S6, 2.2-555.6 μg/mL), and KYQG S1-S11 samples (55.6 μg/mL) exhibited no cytotoxic activity in 24-hour cultures (Table 3). ***Cytokine measurement by enzyme-linked immunosorbent assay (ELISA). Anti-inflammatory effects of KYQG S6:***The production of four pro-inflammatory cytokines increased significantly in the model group (*P* < 0.05, *P* < 0.01). After treatment, TNF-α, IL-8 and IL-1β levels decreased significantly in the Dex (1, 10 μM), NP (10 μg/mL), and KYQG S6 (5.6, 55.6, 555.6 μg/mL) groups (*P* < 0.05, *P* < 0.01). IL-6 levels decreased significantly in Dex (10 μM) and KYQG S6 (55.6, 555.6 μg/mL) groups (*P* < 0.05) (Table 4, Fig. 3). ***Anti-inflammatory effects of KYQG S1-S11 samples:*** The production of TNF-α, IL-8, IL-6, and IL-1β increased significantly in the model group (*P* < 0.01). After treatment, Dex was significantly effective at reducing the levels of the four pro-inflammatory cytokines (*P* < 0.05, *P* < 0.01). NP significantly decreased TNF-α, IL-8, and IL-1β levels (*P* < 0.01). For KYQG S1-S11 samples, the contents of TNF-α decreased significantly in the S1, S4, S5, S6, and S11 groups (*P* < 0.05, *P* < 0.01). IL-8 levels decreased significantly in the S5 and S6 groups (*P* < 0.05, *P* < 0.01). IL-6 levels decreased significantly in the KYQG samples with the exception of the S3 and S7 groups (*P* < 0.05, *P* < 0.01). IL-1β levels decreased significantly in the KYQG samples with the exception of the S1, S2, and S7 groups (*P* < 0.05, *P* < 0.01) (Table 5, Fig. 4). These data indicate large differences in the anti-inflammatory parameters among the eleven KYQG samples.

### Bioactive ingredients o**n** ***anti-inflammatory*** effects in KYQG

Correlations among thirty-eight independent variables (identified ingredients) and four dependent variables (anti-inflammatory effects) were analysed to assess the contribution of each ingredient utilizing grey relational analysis (GRA), principal component analysis (PCA), and partial least-squares (PLS) methods (Table 6). ***Calculated results of IL-1β:*** Harpagide (P12), angoroside C (P23), harpagoside (P27), cinnamic acid (P28), and ruscogenin (P37) effectively contributed to the inhibition of IL-1β release. ***Calculated results of TNF-α:*** Chelidonic acid (P8), neochlorogenic acid (P13), chlorogenic acid (P14), cryptochlorogenic acid (P15), luteolin-7-O-glucoside (P19) and 3, 5-dicaffeoylquinic acid (P21) were the major ingredients effective at suppressing TNF-α release. ***Calculated results of IL-8:***L-pyroglutamic acid (P9), neochlorogenic acid (P13), cryptochlorogenic acid (P15), isoquercitrin (P18), and 3, 4-dicaffeoylquinic acid (P20) had the closest relevance to attenuating IL-8 release. ***Calculated results of IL-6:*** Lysine (P1), γ-aminobutyric acid (P6), tyrosine (P10), angoroside C (P23), and ruscogenin (P37) were more closely related to attenuating IL-6 release. In addition, a dynamic KYQG ingredient-effect relevance bubble chart and a dynamic KYQG herb-effect relevance bubble chart based on the computed results are provided as supplementary files (KYQG ingredient-effect relevance bubble chart S1, KYQG herb-effect relevance bubble chart S2) to illustrate a clear picture of the anti-inflammatory effects of the 38 identified ingredients and 5 herbs.

## Discussion

Oral mucosa keratinocytes comprise the main component of the oral mucosa epithelial cells, acting as a major barrier for oral diseases. After stimulation, oral mucosa keratinocytes produce cytokines that are involved in inflammatory processes. In addition, CSE contains a variety of harmful chemicals and causes inflammatory disorders in keratinocytes or epithelial cells, thus inducing the overexpression of pro-inflammatory cytokines, such as tumour necrosis factor-α (TNF-α), interleukin (IL)-1, IL-6, and IL-8^26^,^27^,^28^. Keratinocytes were treated with 5% CSE to generate an *in vitro* inflammation model in the present study.

Some recent evidence suggests that TNF-α, IL-8, IL-6, and IL-1β are notable mediators of inflammation, playing a prominent part in the development and progression of inflammatory disorders^29^. TNF-α induces the production of inflammatory mediators (e.g., IL-1, IL-6, etc.), promoting inflammatory cell migration. IL-8 possesses diverse functions, participating in the activation of neutrophils and the chemotaxis of neutrophils, T cells, and basophils. IL-6 regulates the proliferation, differentiation, and migration of inflammatory cells. IL-1β induces the expression of adhesion molecules and enhances leukocyte migration^30^,^31^,^32^. Recurrent aphthous ulcers, oral mucositis, oral lichen planus and other oral inflammatory disorders are generally associated with parasecretion of various pro-inflammatory cytokines (e.g., TNF-α, IL-1β, IL-6, IL-8, etc.)^33^,^34^,^35^,^36^,^37^. Thus, the modulation of pro-inflammatory cytokine production is conducive to recovery from oral inflammatory disorders. The KYQG S6 (original formula) results revealed that the alleviation of oral inflammatory disorders by the Chinese herbal formula KYQG is potentially related to the regulation of TNF-α, IL-8, IL-6, and IL-1β.

Mathematically, the relevance analysis of UFLC-DAD-Q-TOF-MS/MS characters and bioactive effects is an assessment of correlations between variables. Numerous mathematical statistical methodologies are available, and every method has its own theoretical basis and scope of application. Thus, the combination of several suitable methods is necessary to comprehensively and truly reflect the information from the experimental data. GRA’s basic ideology is to estimate the associated degree of factors by studying the change in their similarity. The method is applicable to problems with complicated interrelationships among multiple factors and variables and is feasible in multiple attribute decision^38^,^39^. With the GRA method, the degree of association between each ingredient and bioactive effects is visually presented. However, multicollinearity problems can occur among the 38 original independent variables only using GRA. Thus, PCA and PLS were employed. PCA can reassemble the original variables into several mutually independent variables, and then some of the latter variables may be taken to reflect the original variables as much as possible^40^. In this study, five principal components were extracted from the original variables, contributing to 94.36% of the total variance. The scores between five principal components and the original variables were computed for their conversion (Table 7). PLS regression initiated by S. Wold and C. Albano is a novel multivariate statistical approach with the merits of overcoming the multicollinearity problem among the independent variables, discriminating between system signals and noises, and explaining dependent variables. Furthermore, the technique can integrate three analytical approaches: multiple linear regression, PCA, and typical correlation analysis. Overall, the combination of GRA, PLS, and PCA methods are helpful to reveal the relevance of ingredients and bioactive effects.

Organic acids and flavonoids are the main effective ingredients in FL^41^. Neochlorogenic acid (P13), chlorogenic acid (P14), cryptochlorogenic acid (P15), 3,4-dicaffeoylquinic acid (P20), and 3,5-dicaffeoylquinic acid (P21) are organic acids. Isoquercitrin (P18) and luteolin-7-O-glucoside (P19) are flavonoids. Neochlorogenic acid (P13) reportedly inhibits H_2_O_2_ and TNF-α-induced IL-8 secretion *in vitro*^42^. Chlorogenic acid (P14) attenuates the production of TNF-α^43^, and 3,4-dicaffeoylquinic acid (P20) suppresses prostaglandin E_2_ release^44^. In addition, 3,5-dicaffeoylquinic acid (P21) inhibits TNF-α gene expression in lipopolysaccharide (LPS)-stimulated cells^45^. Isoquercitrin (P18) suppresses the release of macrophage inflammatory protein-2 and prostaglandin E_2_ *in vivo*^46^, and luteolin-7-glucoside (P19) reduces LPS-induced TNF-α release from mouse macrophages^47^. Computed results in this study, which are consistent with these reports, demonstrate that the 5 organic acids and 2 flavonoids noted above are anti-inflammatory ingredients in KYQG.

Harpagide (P12), angoroside C (P23), harpagoside (P27), and cinnamic acid (P28) are the bioactive components in RS that exert anti-inflammatory activities as evidenced by numerous *in vivo* and *in vitro* reported experiments. Harpagoside (P27) prevents IL-1β production by RAW 264.7^48^. Harpagide (P12) possesses anti-inflammatory activity in carrageenan-induced inflammation *in vivo*^49^. Angoroside C (P23) exhibits activity in the regulation of nitric oxide (NO) in LPS-stimulated macrophages^50^. Cinnamic acid (P28) reduces LPS-stimulated IL-6 production in macrophages. These 4 ingredients exhibit a close relevance to anti-inflammatory effects and are also the active ingredients in KYQG.

Steroidal saponins are the main bioactive compounds in RO that perform anti-inflammatory, anti-aging, anti-tumour, and immunomodulatory activities. Ruscogenin (P37) is a steroidal saponin. Previous research has demonstrated that ruscogenin (P37) can inhibit LPS-induced lung inflammation by down-regulating intercellular adhesion molecule-1 and suppressing NF-κB activation in anti-inflammatory pathways^51^,^52^. The high levels of chelidonic acid (P8) in RA can reduce dextran sulphate sodium-induced TNF-α production^53^. Our results indicated that ruscogenin (P37) might have positively contribute to the inhibition of IL-1β and IL-6, and chelidonic acid (P8) potentially positively contribute to the suppression of TNF-α. Thus, ruscogenin (P37) and chelidonic acid (P8) are considered to be the anti-inflammatory ingredients in KYQG.

Amino acids, such as lysine (P1) and γ-aminobutyric acid (P6), potentially accelerate wound healing and enhance immune ability^54^,^55^. γ-aminobutyric acid (P6) reportedly suppresses IL-6 release^56^, and tyrosine (P10) significantly decreases gelatine-induced inflammation^57^. Consistent with these literatures, the calculated results demonstrate that several amino acids, including lysine (P1), γ-aminobutyric acid (P6), and tyrosine (P10), positively contribute to the suppression of IL-6 or IL-8. These amino acids are the anti-inflammatory ingredients found in KYQG.

In addition, several saponins exerted negative effects on the following anti-inflammatory parameters: macranthoidin B (P29) and macranthoside A (P30) against IL-1β; saponin 2 (P26) against IL-8 and TNF-α; dipsacoside B (P31) against IL-6; and ruscogenin (P37) against IL-8). These saponins may exert activities other than the anti-inflammatory parameters detected in the present study, and the interaction among these saponins and other bioactive ingredients should also be investigated. However, the specific pharmacological mechanisms need further exploration and verification.

In summary, a novel approach to define the bioactive ingredients of the anti-inflammatory activities in KYQG was established based on a series of UFLC-DAD-Q-TOF-MS/MS analyses and *in vitro* pharmacological experiments. Our results suggested that one specific proinflammatory cytokine could be affected by two or more bioactive ingredients, and the same bioactive ingredient could positively influence two or more proinflammatory cytokines, reflecting the synergy among the ingredients in the traditional Chinese herbal formula. A conclusion could be drawn based on the relevance analysis of ingredients and effects that lysine (P1), γ-aminobutyric acid (P6), chelidonic acid (P8), tyrosine (P10), harpagide (P12), neochlorogenic acid (P13), chlorogenic acid (P14), cryptochlorogenic acid (P15), isoquercitrin (P18), luteolin-7-O-glucoside (P19), 3,4-dicaffeoylquinic acid (P20), 3,5-dicaffeoylquinic acid (P21), angoroside C (P23), harpagoside (P27), cinnamic acid (P28), and ruscogenin (P37) are the core anti-inflammatory bioactive ingredients in KYQG.

## Methods

### Preparation of samples

The KYQG samples were prepared using a five-factor, eleven-level uniform design, in which eleven KYQG samples were prepared by changing the contents of 5 herbs. Sample 6 (S6) was the original KYQG formula ratio. For each sample, the 5 herbs were decocted twice with water, and the liquids were mixed together and filtered. After concentrating the filtrate, ethanol was added. The mixture was stirred thoroughly and then stood for at least 12 hours. The supernatant was later filtered followed by the recovery of ethanol and evaporation of water. Bioactive *in vitro* experiments were conducted as follows: each KYQG sample was diluted with RPMI-1640 medium. KYQG sample 6 (S6, the original formula) was added to the cultures at final concentration of 5.6, 55.6, and 555.6 μg/mL, and the other KYQG samples were maintained at a concentration of 55.6 μg/mL. After being filtered through a 0.22-μm sterile membrane filter (Millipore, Bedford, MA, USA), the KYQG samples were stored at 4 °C until required. The 5 herbs (batch No: 140815) were provided by Hutchison Whampoa Guangzhou Baiyunshan Chinese Medicine Co., Ltd. (Guangzhou, China).

### Reagents

Dex, psoralen (National Institute for the Control of Pharmaceutical and Biological Products, Beijing, China); NP (Beijing Tong-ren-tang Technology Development Co., Ltd., Beijing, China); cigarettes (China Tobacco Guangdong Industrial Co., Ltd., Guangzhou, China); RPMI-1640 medium, penicillin, streptomycin (Hyclone Laboratories, Logan, UT, USA); and foetal bovine serum (FBS) (Gibco, USA) were used in this study. ELISA kits for tumour necrosis factor-α (TNF-α), interleukin-8 (IL-8), interleukin-6 (IL-6), and interleukin-1β (IL-1β) (USCN Life Science, Wuhan, China) were employed. In addition, 3-(4,5-dimethylthiazol-2-yl)-2,5-diphenyl tetrazolium bromide (MTT) (Sigma, USA) was also utilized.

### UFLC-DAD-Q-TOF-MS/MS analysis

Analyses of the KYQG samples were performed using UFLC-DAD-Q-TOF-MS/MS, an ultra-fast liquid chromatography (Shimadzu, Japan) coupled with an SPD-M20A diode array detector (DAD; Shimadzu, Japan) and quadrupole/time-of-flight mass spectrometry (Triple TOF 5600 mass spectrometer, AB SCIEX, USA). Each KYQG sample was suspended in 10 mL of methanol/purified water (50:50, v/v) and filtered through a 0.22-μm membrane, with 100 μL psoralen [52.24 μg/mL in methanol/purified water (50:50, v/v)] added as an internal standard. Then, the sample was diluted with methanol/purified water (50:50, v/v) to a final concentration of 0.15 g/mL for analysis. Gradient chromatographic separation was performed on a Dionex Bonded Silica C_18_ column (3 μM, 150 mm × 4.6 mm) and operated at 40 °C. The injection volume was 5 μL, and the flow rate was 0.3 mL/min. The mobile phase consisted of acetonitrile containing 0.1% formic acid (A) and water containing 0.1% formic acid (B). The elution program was 0 to 7 min (98–90% B), 7 to 95 min (90–59% B), 95 to 105 min (59-0%B), and 105 to 115 min (0%B). The DAD scanned from 190 nm to 400 nm. Electrospray ionization (ESI) source was operated in the positive ion mode with the following settings: ion spray voltage  =  5500 V, source gas 1 = 55 psi, source gas 2 = 55 psi, ion source heater temperature = 550 °C, curtain gas setting = 35 psi, and collision gas pressure = 10 psi. Nitrogen was used as a nebulizer and auxiliary gas. The ingredients in KQYG were identified or tentatively characterized according to the mass spectrum data, related literature, and reference substances. To assess the difference among the prepared eleven KYQG samples, the relative peak areas of identified ingredients calibrated by psoralen (internal standard) were entered into SPSS 19.0 (SPSS, Inc., Chicago, IL, USA) to conduct a cluster analysis. Between-group linkage as the amalgamation rule was applied in this study, whereas Pearson’s correlation was employed to calculate distance.

### Evaluation ***of anti-inflammatory activi*** ty *in vitro*

#### Cell cultures

Human oral keratinocytes (HOK, Guangzhou Jenniobio Biotechnology Company, Guangzhou, China), were cultured in RPMI-1640 medium supplemented with 10% foetal bovine serum, 100 U/mL penicillin, and 100 μg/mL streptomycin. All cells were grown in a 37 °C incubator (5% CO_2_, 95% air) and passaged at approximately 90% confluence. All assays using HOK were performed within the 10th passage in 24- or 96-well plates. ***Preparation of cigarette smoke extract.***CSE was prepared as described previously^58^,^59^,^60^ with the following modifications. Two cigarettes were combusted to produce 300 mL smoke, which was then sucked into a syringe containing 10 mL RPMI-1640 medium. After fully dissolved, the resulting suspension was filtered through a 0.22-μm filter, and this solution was defined as 100% CSE. Then, the desired concentrations of CSE (1%, 2%, 3%, 4%, 5%, 10%, 15%, 20%) were obtained by further diluting 100% CSE with RPMI-1640 medium within 30 min. ***Cell Viability Assay.*** HOK (5 × 10^3^ cells/well) were cultured for 24 hours in 96-well plates and treated with various concentrations of CSE, Dex, NP, eleven KYQG samples for another 24 hours. Then, the MTT assay was conducted to assess the cell cytotoxicity. ***Cytokine measurement by ELISA.*** HOK (2 × 10^5^ cells/well) were cultured for 24 hours in 24-well plates and then serum-starved overnight. For experiments with KYQG S6 (the original formula), the cells were divided randomly into 8 groups as follows: control, model, Dex (1, 10 μM), NP (10 μg/mL), and KYQG S6 (5.6, 55.6, 555.6 μg/mL). For experiments with all the eleven samples, the cells were divided randomly into the following 15 groups: control, model, Dex (1 μM), NP (10 μg/mL), and KYQG S1-S11 samples (55.6 μg/mL). Different groups were pre-incubated with drugs or RPMI-1640 medium for 1 hour followed by an additional 24 hours with 5% of CSE (tested to exhibit no cytotoxic activity by MTT assay) or the same volume of RPMI-1640 medium. TNF-α, IL-8, IL-6 and IL-1β levels in the cell culture supernatants were determined with ELISA kits.

### Mathematical methods of relevance analysis

#### Data dimensionless

Relative peak areas of identified ingredients were regarded as independent variables (Tables 8 and 9), and values of anti-inflammatory effects were regarded as dependent variables (Tables 10 and 11). Negative effect values (the smaller the better) were converted to positive values by taking the reciprocal. All of the relative peak areas and effect values were then processed to be dimensionless by the equalization method for the relevance analysis. ***Grey relational analysis (GRA)***. GRA is a method of measuring the degree of influence between each given comparative series and the reference series in a system^61^, and this degree of influence is referred to the grey relational degree (GRD). In this study, ingredient variables were taken as the comparative series, and the effect values (TNF-α, IL-8, IL-6 and IL-1β) were the reference series. GRD was calculated with a distinguishing coefficient of 0.5^61^. The higher the GRD, the greater the effect of corresponding ingredient. ***Principal component analysis (PCA)***. PCA is a popular technique for dimension reduction of multivariate problems^62^. All of the ingredient variables were simplified to a few new independent variables, which are mutual independent principal components. The regression equations between new principal components and effect values (TNF-α, IL-8, IL-6 and IL-1β) were established with a stepwise regression method. Once a regression equation was formed (*P* < 0.05, IL-6 and IL-1β), the original independent variables would replace the new principal components to establish another proper multilinear regression equation. Regression coefficients (RCs) were used to estimating the effect contribution of each ingredient. When no available equations (*P* > 0.05) were formed (IL-8 and TNF-α), Pearson’s correlation coefficients (PCCs) between the original independent variables and effect values were computed directly. In the present study, the regression equation between extracted components and IL-1β was used, as follows: Y_IL-1β_ = 0.066 + 0.003 × Component4-0.001 × Component1 (*P* < 0.05). After replacing the extracted components with the original variables, we obtain the following equation: Y_IL-1β_ = 0.066  + 3.51 × 10^−4^ P1 + 6.10 × 10^−4^ P2 + ……+ 7.91 × 10^−4^ P38. The regression equation between extracted components and IL-6 is noted as follows: Y_IL-6_ = 0.401 + 0.013 × Component4+0.008 × Component2 (*P* < 0.05). After replacing the extracted components with the original variables, we obtain the following equation: Y_IL-6_ = 0.401 + 3.84 × 10^−3^ P1 + 4.15 × 10^−3^ P2+……+1.78 × 10^−3^ P38. ***Partial least-squares (PLS) regression***. PLS regression effectively settles the multicollinearity problem among variables and is suitable for situations in which the number of observations is less than the number of variables^63^. In this study, the standardized regression coefficients (RCs) in PLS regression between the independent variables and effect values (TNF-α, IL-8, IL-6 and IL-1β) were calculated to evaluate every ingredient’s effect contribution. All of the processes above were implemented in SPSS 19.0.

### Statistical analysis

Data are presented as the mean ± standard deviation (S.D.) and processed by one-way analysis of variance (ANOVA), Student’s t tests, and least significant difference multiple comparisons in SPSS 19.0. *P*-values less than 0.05 or 0.01 were considered to be statistically significant.

## Additional Information

**How to cite this article**: Liu, H. *et al.* Discovery of Anti-inflammatory Ingredients in Chinese Herbal Formula Kouyanqing Granule based on Relevance Analysis between Chemical Characters and Biological Effects. *Sci. Rep.* **5**, 18080; doi: 10.1038/srep18080 (2015).

## Supplementary Material

Supplementary Information

Supplementary Information

Supplementary Information

## Figures and Tables

**Table 1:** Weights and percentage of 5 herbs in the eleven KYQG samples under a uniform design.

	**FL (g-%)**	**RS (g-%)**	**RA (g-%)**	**RO (g-%)**	**RG (g-%)**
S1	846.0−36.0	0−0	394.8−16.8	592.2−25.2	517.0−22.0
S2	799.0−34.0	98.7−4.2	888.3−37.8	98.7−4.2	465.3−19.8
S3	752.0−32.0	197.4−8.4	197.4−8.4	789.6−33.6	413.6−17.6
S4	705.0−30.0	296.1−12.6	690.9−29.4	296.1−12.6	361.9−15.4
S5	658.0−28.0	394.8−16.8	0−0	987.0−42.0	310.2−13.2
S6	611.0−26.0	493.5−21.0	493.5−21.0	493.5−21.0	258.5−11.0
S7	564.0−24.0	592.2−25.2	987.0−42.0	0−0	206.8−8.8
S8	517.0−22.0	690.9−29.4	296.1−12.6	690.9−29.4	155.1−6.6
S9	470.0−20.0	789.6−33.6	789.6−33.6	197.4−8.4	103.4−4.4
S10	423.0−18.0	888.3−37.8	98.7−4.2	888.3−37.8	51.7−2.2
S11	376.0−16.0	987.0−42.0	592.2−25.2	394.8−16.8	0−0

FL = Flos lonicerae; RS = Radix scrophulariae; RO = Radix ophiopogonis; RA = Radix asparagi; RG = Radix et rhizoma glycyrrhizae.

**Table 2:** Identification details of the ingredients in KYQG represented by 38 peaks using a UFLC-Q-TOF-MS/MS technique.

**Variables**	**Retention time (min)**	**Attribution^a^**	**Ingredients**	**Molecular formula**	**[M + H]**^**+**^	**[M − H]**^**−**^	**Positive mode**	**Negative mode**
P1	4.92	AL	Lysine^b^	C_6_H_14_N_2_O_2_	147.1128 (−0.4)		130.0867[M+H-NH_3_]^+^, 84.0826[M+H-NH_3_-HCOOH]^+^, 67.0566[M+H-2NH_3_-HCOOH] ^+^	
P2	5.27	AL	Arginine^b^	C_6_H_14_N_4_O_2_	175.1190 (0)	173.1058 (8.1)	158.0934[M+H-NH_3_]^+^, 130.0985[M+H-COOH]^+^, 70.681[M+H- NH_3_-C_3_H_6_NO_2_]^+^, 60.0594[M+H-C_5_H_10_NO_2_]^+^	156.0767[M-H-NH_3_]^−^, 131.0827[ M-H-C_3_H_6_]^−^
P3^17^,^19^	5.32	AL	Asparagine	C_4_H_8_N_2_O_3_	133.0606 (−1.4)		116.0336[M+H-NH_3_]^+^, 87.0565[M+H-HCOOH]^+^, 74.0259[M+H-C_2_H_5_NO]^+^, 70.0312[M+H-HCOOH-NH_3_]^+^	
P4	5.36	AL	Aspartic acid^b^	C_4_H_7_NO_4_	134.0451 (0.3)	132.0315 (1.2)	116.0338[M+H-H_2_O]+, 88.0404[M+H-HCOOH]^+^, 74.0256[M+H-NH_3_-CO-CH_3_]^+^	115.0045[M-H-NH_3_]^−^, 88.0413[M-H-CO_2_]^−^, 71.0154[M-H- CO_2_-NH_3_]^−^
P5	5.59	AL	Citrulline^b^	C_6_H_13_N_3_O_3_	176.1029 (−0.5)		159.0765[M+H-NH_3_]^+^, 113.0709[M+H-NH_3_-HCOOH]^+^, 70.0673[M+H-NH_3_-CO_2_-CH_3_NO]^+^	
P6^18^	5.66	AL	γ-aminobutyric acid	C_4_H_9_NO_2_	104.0706 (−0.1)		87.0457[M+H-NH_3_]^+^, 69.0359[M+H-NH_3_-H_2_O]^+^, 60.0835[M+H-CO_2_]^+^	
P7^19^	6.05	AL	Proline	C_5_H_9_NO_2_	116.0709 (0.8)		70.0677[M+H-HCOOH]^+^, 68.0521[M+H-HCOOH-H_2_O]^+^	
P8	6.61	RA/RO	Chelidonic acid^b^	C_7_H_4_O_6_	185.0082 (0.7)	182.9939 (1.8)	141.0173[M+H-CO_2_]^+^, 97.0297[M+H-2CO_2_]^+^, 71.0152[M+H-2CO_2_-C_2_H_2_]^+^	139.0038[M-H-CO_2_]^−^, 68.9988[M-H-C_4_H_2_O_4_]^−^, 67.0207[M-H-2CO2-CO]^−^
P9^20^	10.20	AL	Pyroglutamic acid	C_5_H_7_NO_3_	130.0500 (1.1)		84.0461[M+H-HCOOH]^+^, 56.0531[M+H-HCOOH-CO]^+^	
P10	11.13	AL	Tyrosine^b^	C_9_H_11_NO_3_	182.0811 (−0.2)		165.0543[M+H-NH_3_]^+^, 147.0435[M+H-NH_3_-H_2_O]^+^, 136.0755[[M+H-HCOOH]^+^, 119.0490[[M+H-HCOOH-NH_3_]^+^, 91.0553[M+H-HCOOH-NH_3_-CO]^+^, 77.0403[M+H-HCOOH-NH_3_-CO-CH_2_]^+^, 65.04111[M+H-HCOOH-NH_3_-CO-C_2_H_2_]^+^	
P11	15.28	AL	Phenylalanine^b^	C_9_H_11_NO_2_	166.0864 (0.9)	164.0725 (4.6)	120.0816[M+H-HCOOH]^+^, 103.0554[M+H-HCOOH-NH_3_]^+^	165.0760[M], 147.0445[M-H-NH_3_]^−^, 103.0555[M-H-NH_3_-CO_2_]^−^, 72.0096[M-H-NH_3_-C_6_H_5_]^−^
P12	15.35	RS	Harpagide^b^	C_15_H_24_O_10_		363.1282 (−1.2)	387.1256[M+Na]^+^	409.1338[M+COOH]^−^, 201.0760[M-H-Glc]^−^, 183.0656[M-H-Glc-H_2_O]^−^, 165.0549[M-H-Glc-H_2_O-H_2_O]^−^, 139.0388[M-H-Glc-H_2_O-H_2_O-C_2_H_2_]^−^
P13 21	18.38	FL	Neochlorogenic acid	C_16_H_18_O_9_	355.1025 (−1.9)	353.0877 (−0.8)	163.0396[M+H- C_7_H_12_O_6_]^+^, 145.0288[M+H- C_7_H_12_O_6_-H2O ]^+^, 117.0342[ M+H-C_7_H_12_O_6_-H2O-CO]^+^, 89.0402[M+H-C_7_H_12_O_6_-H2O-CO-CO]^+^	191.0557[M-H-C_9_H_6_O_3_]^−^, 179.0344[M-H-C_7_H_10_O_5_]^−^, 135.0450[M-H- C_7_H_10_O_5_-CO_2_]^−^
P14	23.65	FL	Chlorogenic acid^b^	C_16_H_18_O_9_	355.1026 (0.6)	353.0891 (−1.3)	163.0394[M+H-C_7_H_12_O_6_]^+^, 145.0290[M+H-C_7_H_12_O_6_-H_2_O]^+^, 117.0344[M+H-C_7_H_12_O_6_-H_2_O-CO]^+^, 89.0405[M+H-C_7_H_12_O_6_-H_2_O-2CO]^+^	191.0561[M-H-C_9_H_6_O_3_]^−^
P15 21	24.69	FL	Cryptochlorogenic acid	C_16_H_18_O_9_	355.1027 (0.9)	353.0869 (−0.5)	163.0390[M+H-C_7_H_12_O_6_]^+^, 145.0284[M+H-C_7_H_12_O_6_-H_2_O]^+^, 117.0338[M+H-C_7_H_12_O_6_-H_2_O-CO]^+^	191.0548[M-H-C_9_H_6_O_3_]^−^, 173.0442[M-H-C_9_H_6_O_3_-H_2_O]^−^, 135.0441[M-H-C_7_H_10_O_5_-CO_2_]^−^, 93.0345[M-H-C_9_H_7_O_4_-2H_2_O-COOH]^−^
P16	28.59	FL	Caffeic acid^b^	C_9_H_8_O_4_	181.0409 (−0.7)	179.0354 (0.2)	163.0387[M+H-H_2_O]^+^, 135.0439[M+H-H_2_O-CO]^+^, 89.3980[M+H-H_2_O-CO-HCOOH]^+^	135.0446[M-H-CO_2_]^−^
P17	42.04	RG	Liquiritin^b^	C_21_H_22_O_9_	419.1334 (−0.7)	417.1189 (−0.6)	257.0803[M+H-Glc]^+^, 137.0224[M+H-C_14_H_18_O_6_]^+^	255.0645[M-H-Glc]^−^, 135.0077[M-H-C_14_H_18_O_6_]^−^, 119.0489[M-H-C_14_H_18_O_6_-Glc]^−^
P18	42.69	FL	Isoquercitrin^b^	C_21_H_20_O_12_	465.1027 (−0.1)	463.0876 (−1.3)	303.0489[M+H-Glc]^+^	301.0351[M-H-Glc]-, 271.0240[M-Glc-CHO]^−^, 255.0285[M-H-Glc-O-CHO]^−^, 151.0016[M-H-C_7_H_3_O_3_]^−^
P19	43.44	FL	Luteolin-7-O-glucoside^b^	C_21_H_20_O_11_	449.1077 (−0.3)	447.0917 (−2.3)	287.0554[M+H-Glc]^+^	285.0404[M-H-Glc]^−^
P20 22	46.33	FL	3,4-Dicaffeoylquinic acid	C_25_H_24_O_12_	517.1335 (−1.0)	515.1178 (−1.3)	499.1231[M+H-H_2_O]^+^, 163.0389[M+H-C_6_H_18_O_9_]^+^, 145.0286[M+H-C_6_H_18_O_9_-H_2_O]^+^	353.0853[M-H-C_9_H_6_O_3_]^−^, 191.0541[M-H-2C_9_H_6_O_3_]^−^, 173.0435[M-H-2C_9_H_6_O_3_-H_2_O]^−^
P21 22	48.29	FL	3,5-Dicaffeoylquinic acid	C_25_H_24_O_12_	517.1325 (−0.6)	515.1195 (−2.5)	499.1229[M+H-H_2_O]^+^, 163.0390[M+H-C_6_H_18_O_9_]^+^, 145.0280[M+H-C_6_H_18_O_9_-H_2_O]^+^	353.0867[M-H-C_9_H_6_O_3_]^−^, 191.0546[M-H-2C_9_H_6_O_3_]^−^, 173.0437[M-H-2C_9_H_6_O_3_-H_2_O]^−^
P22 22	52.11	FL	4,5-Dicaffeoylquinic acid	C_25_H_24_O_12_	517.1336 (2.4)	515.1181 (−3.4)	499.1240[M+H-H_2_O]^+^, 163.0397[M+H-C_16_H_18_O_9_]^+^, 145.0289[M+H-C_16_H_18_O_9_-H_2_O]^+^	353.0853[M-H-C_9_H_6_O_3_]^−^, 191.0541[M-H-2C_9_H_6_O_3_]^−^, 173.0435[M-H-2C_9_H_6_O_3_-H_2_O]^−^
P23	53.51	RS	Angoroside C^b^	C_36_H_48_O_19_		783.2723 (0.7)	807.2675 [M+Na]^+^	607.2276[M-H-C_6_H_11_O_4_-CHO]^−^, 193.0492[M-H-C_26_H_38_O_15_]^−^, 175.0383[M-H-C_26_H_38_O_15_-H_2_O]^−^
P24	59.42	RG	Isoliquiritoside^b^	C_21_H_22_O_9_	419.1334 (−0.7)	417.1177 (−0.6)	257.0812[M+H-Glc]^+^, 137.0224[M+H-Glc-C_8_H_6_O]^+^	255.0648[M-H-Glc]^−^, 119.0489[M-H-Glc-C_8_H_6_O-H_2_O]^−^, 92.0260[M-H-Glc-C_9_H_7_O_3_]^−^
P25 23	63.49	RA	Saponin 1^c^	—	1035.5357		1035.5371, 579.3893,417.3358	
P26 23	65.46	RA	Saponin 2^c^	—	903.4940		903.4922,741.4405,417.3335	
P27	70.83	RS	Harpagoside^b^	C_24_H_30_O_11_		493.1679 (−4.9)	517.1675[M+Na]^+^	539.1745[M+COOH]^−^, 147.0434[M-H-C_15_H_22_O_9_]^−^
P28	73.23	RS	Cinnamic acid^b^	C_9_H_8_O_2_	149.0598 (0)	147.0452 (0.2)	131.0490[M+H-H2O]^+^, 103.0550[M+H-HCOOH]^+^	103.0529[M-COOH]^−^
P29	74.34	FL	Macranthoidin B^b^	C_65_H_106_O_32_		1397.6515 (−3.9)	1421.6554[M+Na]^+^	1443.6582[M+COOH]^−^, 1073.5507[M-H-2Glc]^−^
P30 24	76.63	FL	Macranthoside A	C_47_H_76_O_17_	913.5130 (−1.4)		782.4876[M+H-H_2_O]^+^, 751.4601[M+H-Glc]^+^, 455.3512[M+H- Ara-Rha-Glc]^+^, 437.3398[M+H- Ara-Rha-Glc-H_2_O]^+^, 409.3510[M+H-Ara-Rha-Glc-HCOOH]^+^	
P31	79.46	FL	Dipsacoside B^b^	C_53_H_86_O_22_		1073.5561 (−2.1)	1097.5470[M+Na]^+^	1119.5572[M+COOH]^−^, 749.4483[M-H-2Glc]^−^
P32	99.32	RG	Glycyrrhetic acid^b^	C_30_H_46_O_4_		469.3289 (−3.5)	471.3460[M+Na]^+^	469.3289[M-H]^−^
P33	99.33	RG	Glycyrrhizic acid^b^	C_42_H_62_O_16_	823.4098 (−1.1)		471.3453[M+H-2C_6_H_8_O_6_]^+^, 453.3362[M+H-2C_6_H_8_O_6_-H_2_O]^+^,	
P34 25	105.34	RG	Gancaonin B	C_21_H_20_O_6_	369.1334 (0.3)		285.0765[M+H-C_5_H_8_O]^+^, 270.0525[M+H-C_5_H_6_O_2_]^+^, 255.0648[M+H-C_5_H_6_O_2_-CH_3_]^+^	
P35	108.09	RA	Sarsasapogenin ^b^	C_27_H_44_O_3_	417.3359 (−0.9)	415.3218 (3.9)	417.3353[M+H]^+^, 273.2216 225.2114	
P36	108.92	RO	Methylophiopogonanone A^b^	C_19_H_18_O_6_	343.1170 (−4.1)		207.0644[M+H-C_8_H_8_O_2_]^+^, 135.0436[M+H-C_11_H_12_O_4_]^+^	
P37	109.09	RO	Ruscogenin^b^	C_27_H_4_2O_4_	431.3157 (−0.1)	429.3010 (−2.5)	431.3133[M+H]^+^, 287.2005, 269.1899	
P38	109.09	RO	Ophiopogonin D^b^	C_44_H_70_O_16_	855.4745 (−2.8)		431.3172[M-Man-Xyl-Gal-2O]^+^ 287.2007	

^a^FL = Flos lonicerae; RS = Radix scrophulariae; RO = Radix ophiopogonis; RA = Radix asparagi; RG = Radix et rhizoma glycyrrhizae; AL = 5 herbs.

^b^Identification compared with reference substances.

^c^Sarsasapogenin as aglycone.

^d^P3 17,19, P6 18, P7 19, P9 20, P13 21, P15 21, P20 22, P21 22, P22 22, P25 23, P26 23, P30 24, P34 ^25^ were characterized by MS data and related studies^17^−^25^.

**Table 3:** Cell viability.

**Sample**	**CSE**								**Dex**			
	**1%**	**2%**	**3%**	**4%**	**5%**	**10%**	**15%**	**20%**	**1 μM**	**10 μM**	**100 μM**	**1000 μM**
Cell viability (%)	99.5 ± 2.5	96.1 ± 2.2	95.4 ± 2.0	94.6 ± 3.6	90.8 ± 1.8	36.2 ± 2.1^**^	18.5 ± 0.9^**^	9.2 ± 0.4^**^	99.7 ± 0.7	99.2 ± 1.8	93.5 ± 1.5	34.4 ± 0.7^**^
**Sample**	**NP**			**KYQG (the original formula)**								
	**10 μg/mL**	**100 μg/mL**	**1000 μg/mL**	**2.2 μg/mL**		**22.2 μg/mL**		**222.2 μg/mL**		**555.6 μg/mL**		**2222.2 μg/mL**
Cell viability (%)	91.9 ± 2.4	90.0 ± 1.2	10.5 ± 0.9^**^	97.0 ± 3.1		95.6 ± 3.2		93.2 ± 3.0		90.7 ± 0.8		63.5 ± 0.9^**^
**Sample**	**S1**	**S2**	**S3**	**S4**	**S5**	**S6**	**S7**	**S8**	**S9**	**S10**	**S11**	
Cell viability (%)	91.2 ± 0.7	92.3 ± 1.3	92.6 ± 2.7	90.8 ± 0.6	89.0 ± 2.3	90.3 ± 3.3	90.6 ± 1.9	89.0 ± 4.8	94.7 ± 0.6	91.4 ± 1.8	88.2 ± 1.8	

Data are shown as the mean ± SD, n = 3.

S1-S11 represented the eleven KYQG samples.

Cell viability in HOK after treatment with CSE, Dex, NP, KYQG (the original formula) and eleven KYQG samples (S1-S11) for 24 hours.

CSE: cigarette smoke extract; Dex: Dexamethasone; NP: Niuhuangjiedu Pian; KYQG: Kouyanqing Granule.

The cell viability of control group was defined as 100%. ^*^P < 0.05 and ^**^P < 0.01 vs. control group.

**Table 4:** The effects of KYQG (the original formula) on IL-1β, TNF-α, IL-8, and IL-6 production.

**Group**	**Dose**	**IL-1β (pg/mL)**	**TNF-α (pg/mL)**	**IL-8 (pg/mL)**	**IL-6 (pg/mL)**
Control	Medium	13.12 ± 0.68	2.56 ± 0.25	463.67 ± 28.62	7.01 ± 0.22
Model	Medium	27.23 ± 2.78^**^	3.42 ± 0.36^*^	745.62 ± 15.17^**^	10.44 ± 0.94^*^
NP	10 μg/mL	16.59 ± 2.29^##^	2.30 ± 0.26^##^	517.02 ± 20.99^##^	11.32 ± 1.40
Dex	1 μM	18.14 ± 1.91^##^	2.10 ± 0.23^##^	555.48 ± 84.21^##^	9.21 ± 0.50
Dex	10 μM	15.51 ± 3.33^##^	1.38 ± 0.04^##^	555.34 ± 54.10^##^	8.16 ± 0.45^#^
KYQG	5.6 μg/mL	19.22 ± 2.72^##^	1.81 ± 0.27^##^	607.59 ± 16.54^#^	9.39 ± 0.78
KYQG	55.6 μg/mL	18.62 ± 0.45^##^	1.56 ± 0.26^##^	551.73 ± 50.41^##^	8.59 ± 0.57^#^
KYQG	555.6 μg/mL	9.65 ± 2.20^##^	0.99 ± 0.26^##^	521.55 ± 36.88^##^	8.16 ± 0.25^#^

Data are presented as the mean ± SD, n = 3.

Dex: Dexamethasone; NP: Niuhuangjiedu Pian; KYQG: Kouyanqing Granule.

Control group and model group received the same volume of RPMI-1640 medium for the treatment.

*P < 0.05 and ^**^P < 0.01 vs. control group, ^#^*P* < 0.05 and ^##^*P* < 0.01 vs. model group.

**Table 5:** The effects of eleven KYQG samples on IL-1β, TNF-α, IL-8, and IL-6 production.

**Group**	**Dose**	**IL-1β (pg/mL)**	**TNF-α (pg/mL)**	**IL-8 (pg/mL)**	**IL-6 (pg/mL)**
Control	Medium	11.63 ± 0.93	1.20 ± 0.02	215.11 ± 9.52	2.14 ± 0.05
Model	Medium	20.28 ± 2.12^**^	1.74 ± 0.03^**^	272.93 ± 12.80^**^	3.14 ± 0.11^**^
NP	10 μg/mL	10.64 ± 0.91^##^	1.23 ± 0.09^##^	239.81 ± 14.04^##^	2.76 ± 0.16
Dex	1 μM	15.21 ± 1.15^##^	1.37 ± 0.02^##^	230.85 ± 3.72^##^	2.60 ± 0.27^#^
S1	55.6 μg/mL	18.92 ± 0.35	1.15 ± 0.10^##^	289.76 ± 3.37	2.37 ± 0.14^##^
S2	55.6 μg/mL	18.06 ± 2.73	1.64 ± 0.19	265.53 ± 13.31	2.53 ± 0.19^##^
S3	55.6 μg/mL	15.83 ± 1.21^##^	1.58 ± 0.12	261.08 ± 14.86	2.87 ± 0.34
S4	55.6 μg/mL	14.72 ± 1.57^##^	1.22 ± 0.07^##^	341.33 ± 14.79	2.53 ± 0.16^##^
S5	55.6 μg/mL	15.96 ± 0.87^##^	1.50 ± 0.12^#^	250.25 ± 6.24^#^	2.64 ± 0.19^#^
S6	55.6 μg/mL	12.62 ± 1.15^##^	1.12 ± 0.14^##^	237.00 ± 10.85^##^	2.53 ± 0.16^##^
S7	55.6 μg/mL	17.44 ± 0.93	1.93 ± 0.11	252.69 ± 9.45	2.79 ± 0.30
S8	55.6 μg/mL	13.11 ± 1.40^##^	1.77 ± 0.02	341.92 ± 7.89	2.14 ± 0.14^##^
S9	55.6 μg/mL	14.23 ± 1.15^##^	1.77 ± 0.08	288.33 ± 8.85	2.56 ± 0.14^##^
S10	55.6 μg/mL	14.35 ± 1.39^##^	1.89 ± 0.11	259.30 ± 2.87	2.60 ± 0.22^#^
S11	55.6 μg/mL	13.61 ± 1.32^##^	1.40 ± 0.15^##^	279.84 ± 15.76	2.11 ± 0.14^##^

Data are presented as the mean ± SD, n = 3.

S1-S11 represented the eleven KYQG samples.

Dex: Dexamethasone; NP: Niuhuangjiedu Pian; KYQG: Kouyanqing Granule.

Control group and model group received the same volume of RPMI-1640 medium for the treatment.

*P < 0.05 and **P < 0.01 vs. control group, ^#^*P* < 0.05 and ^##^*P* < 0.01 vs. model group.

**Table 6:** Calculated results of the relevance between 38 ingredients and 4 effect parameters.

**Ingredients**	**IL-1β**			**TNF-α**			**IL-8**				**IL-6**	
	**GRD**	**PCA-RC** × **10**^**−4**^	**PLS-RC**	**GRD**	**PCC**	**PLS-RC**	**GRD**	**PCC**	**PLS-RC**	**GRD**	**PCA-RC** × **10**^**−3**^	**PLS-RC**
P1	0.703	3.512	−0.002	0.726	0.303	0.010	0.649	−0.278	−0.002	0.734	3.843	0.134
P2	0.710	6.102	0.044	0.729	0.342	0.015	0.669	−0.256	0.009	0.733	4.154	0.057
P3	0.716	−3.302	−0.109	0.750	0.300	−0.026	0.692	−0.263	−0.135	0.733	0.045	0.154
P4	0.690	−3.064	−0.005	0.744	0.522	0.030	0.713	−0.184	−0.165	0.701	−0.384	0.059
P5	0.625	−4.917	−0.214	0.617	−0.125	−0.092	0.596	0.072	0.485	0.626	0.892	−0.117
P6	0.718	3.057	−0.051	0.742	0.377	0.028	0.663	−0.218	0.069	0.729	3.059	0.098
P7	0.661	−0.825	−0.038	0.726	0.551	0.042	0.664	−0.160	−0.158	0.667	0.126	0.093
P8	0.830	−1.948	0.002	0.890	0.654	0.104	0.835	−0.115	−0.415	0.860	0.727	0.287
P9	0.805	1.358	−0.277	0.845	0.395	0.010	0.845	0.131	0.243	0.830	0.611	0.113
P10	0.771	4.040	0.098	0.798	0.572	0.067	0.733	−0.170	−0.180	0.785	3.997	0.138
P11	0.763	2.553	0.027	0.781	0.499	0.030	0.732	−0.172	−0.072	0.782	2.999	0.098
P12	0.716	4.295	0.164	0.693	−0.253	−0.039	0.705	−0.224	0.039	0.751	4.582	0.066
P13	0.797	4.204	−0.016	0.843	0.680	0.087	0.813	0.240	0.161	0.804	2.687	0.005
P14	0.797	1.909	0.088	0.865	0.683	0.089	0.817	0.237	0.033	0.802	1.170	−0.009
P15	0.797	3.016	0.015	0.851	0.649	0.079	0.813	0.276	0.158	0.802	1.988	−0.033
P16	0.786	2.051	−0.037	0.792	0.434	0.003	0.773	−0.050	0.136	0.782	2.885	0.010
P17	0.664	−3.325	0.028	0.736	0.526	0.021	0.691	0.043	−0.084	0.659	−1.445	−0.035
P18	0.737	0.453	0.046	0.811	0.580	0.038	0.755	0.104	0.106	0.735	1.107	−0.058
P19	0.772	−0.179	0.075	0.855	0.662	0.073	0.795	0.186	0.005	0.774	0.268	−0.017
P20	0.809	2.948	−0.022	0.858	0.590	0.054	0.822	0.233	0.220	0.817	2.285	−0.053
P21	0.798	1.100	0.116	0.870	0.703	0.084	0.802	0.081	−0.150	0.800	0.956	0.051
P22	0.811	0.277	0.028	0.873	0.620	0.055	0.823	0.170	0.037	0.813	0.684	−0.011
P23	0.799	7.737	0.271	0.724	−0.263	0.021	0.726	−0.243	−0.308	0.773	4.621	0.098
P24	0.663	−3.070	0.062	0.718	0.620	0.059	0.634	0.038	−0.186	0.655	−1.741	0.010
P25	0.635	−4.924	0.030	0.645	0.109	−0.015	0.613	−0.167	−0.221	0.638	0.707	0.151
P26	0.646	−4.169	−0.107	0.619	−0.513	−0.141	0.604	−0.345	−0.356	0.642	0.497	0.134
P27	0.788	7.508	0.169	0.746	−0.254	0.003	0.734	−0.057	−0.013	0.768	4.759	−0.022
P28	0.707	4.293	0.073	0.645	−0.434	0.010	0.638	−0.017	−0.102	0.706	1.693	0.099
P29	0.756	−8.238	−0.094	0.832	0.120	−0.084	0.781	−0.233	−0.044	0.779	−2.866	−0.136
P30	0.816	−8.395	−0.138	0.859	−0.115	−0.124	0.848	−0.317	0.057	0.845	−2.066	−0.184
P31	0.840	−8.689	−0.068	0.861	−0.129	−0.111	0.872	−0.411	−0.177	0.874	−3.022	−0.151
P32	0.664	−3.409	0.068	0.727	0.546	0.033	0.677	0.006	−0.161	0.660	−1.698	−0.073
P33	0.648	−6.697	0.010	0.711	0.340	−0.031	0.675	−0.095	−0.142	0.670	−2.782	−0.124
P34	0.678	−4.975	−0.014	0.745	0.531	0.030	0.680	0.111	−0.035	0.672	−2.554	−0.038
P35	0.690	−7.085	−0.040	0.673	−0.038	−0.011	0.646	0.083	0.066	0.663	−0.950	0.016
P36	0.740	10.915	0.020	0.722	−0.055	0.060	0.743	0.082	−0.031	0.720	2.529	0.081
P37	0.714	8.958	0.254	0.647	−0.268	0.003	0.657	−0.453	−0.624	0.683	3.595	0.378
P38	0.666	7.906	−0.036	0.718	−0.002	0.068	0.683	0.082	0.186	0.680	1.779	0.002

For the results of grey relational degree (GRD), Pearson's correlation coeffiecient (PCC), and regression coefficient (RC), positive or negative values represent positive or negative effect contributions, respectively. The larger the absolute value, the higher the positive or negative effect contribution.

P12, P23, P27, P28, and P37 mainly contributed to IL-1β; P8, P13, P14, P15, P19, and P21 to TNF-α; P9, P13, P15, P18, and P20 to IL-8; P1, P6, P10, P23, and P37 to IL-6.

P29 and P30 might have a negative effect on IL-1β; P26 on TNF-α; P26 and P37 on IL-8; P31 on IL-6.

Lysine (P1), γ-aminobutyric acid (P6), chelidonic acid (P8), tyrosine (P10), harpagide (P12), neochlorogenic acid (P13), chlorogenic acid (P14), cryptochlorogenic acid (P15), isoquercitrin (P18), luteolin-7-O-glucoside (P19), 3,4-dicaffeoylquinic acid (P20), 3,5-dicaffeoylquinic acid (P21), angoroside C (P23), harpagoside (P27), cinnamic acid (P28) and ruscogenin (P37) are the core bioactive ingredients on anti-inflammatory effects.

The regression equation between the extracted components and IL-1β (stepwise regression): YIL-1β = 0.066 + 0.003 × Component4-0.001 × Component1 (P < 0.05). After replacing the extracted components with the original variables: YIL-1β = 0.066 + 3.51 × 10^−4^P1 + 6.10 × 10^−4^P2+……+7.91 × 10^−4^P38.

The regression equation between extracted components and IL-6 (stepwise regression): YIL-6 = 0.401 + 0.013 × Component4 + 0.008 × Component2 (P < 0.05). After replacing the extracted components with the original variables: YIL-6 = 0.401 + 3.84 × 10^−3^P1 + 4.15 × 10^−3^P2+……+1.78 × 10^−3^P38.

A dynamic KYQG ingredient-effect relevance bubble chart and a dynamic KYQG herb-effect relevance bubble chart based on the computed results are provided as the Supplementary File (KYQG ingredient-effect relevance bubble chart S1, KYQG herb-effect relevance bubble chart S2) to illustrate a clear picture of anti-inflammatory effects of the 38 identified ingredients and 5 herbs.

**Table 7:** The scores of 5 principal components (extracted from 38 ingredient variables).

	**P1**	**P2**	**P3**	**P4**	**P5**	**P6**	**P7**	**P8**	**P9**	**P10**	**P11**	**P12**	**P13**	**P14**	**P15**	**P16**	**P17**	**P18**	**P19**
Component 1	0.1345	0.1400	0.1781	0.2027	0.0751	0.1437	0.1966	0.1654	0.1580	0.1768	0.1907	−0.0509	0.1846	0.1807	0.1789	0.1942	0.2029	0.2070	0.1942
Component 2	0.2173	0.1129	0.0881	0.0082	0.3371	0.1390	−0.0461	0.1068	−0.0828	0.1851	0.1333	0.3677	0.0082	−0.0550	−0.0118	0.1443	−0.1105	0.0016	−0.0620
Component 3	0.2265	0.2561	0.1993	0.1502	−0.0672	0.2704	0.1807	−0.0691	0.0815	0.0219	0.0992	−0.0019	−0.1664	−0.2337	−0.2217	0.0343	−0.0591	−0.0925	−0.1912
Component 4	0.1619	0.2501	−0.0507	−0.0346	−0.1389	0.1498	0.0380	−0.0098	0.0980	0.1936	0.1487	0.1262	0.2017	0.1239	0.1602	0.1331	−0.0432	0.0841	0.0588
Component 5	−0.0756	0.1121	−0.1443	−0.0791	−0.1173	−0.1469	−0.1104	−0.4302	−0.3572	0.0122	0.0330	0.1052	−0.0461	0.0626	0.0130	0.0643	0.1278	0.1460	0.0591
	P20	P21	P22	P23	P24	P25	P26	P27	P28	P29	P30	P31	P32	P33	P34	P35	P36	P37	P38
Component 1	0.1874	0.1994	0.1988	−0.1393	0.1964	0.0827	−0.0359	−0.1147	−0.2064	0.1722	0.1256	0.1014	0.1974	0.1927	0.1933	0.0085	−0.1167	−0.1250	−0.0940
Component 2	0.0245	−0.0481	−0.0371	0.2340	−0.1578	0.3102	0.3074	0.2503	0.0909	−0.0053	0.1284	0.0379	−0.1345	−0.0893	−0.1545	0.2605	−0.2120	0.0318	−0.1549
Component 3	−0.1669	−0.1488	−0.1717	−0.0358	−0.0486	−0.1278	−0.0525	−0.1178	−0.0711	0.2036	0.2689	0.3238	−0.0334	0.0339	−0.0758	−0.2542	0.1078	0.1760	0.2303
Component 4	0.1608	0.1031	0.0755	0.2115	−0.0369	−0.1366	−0.1510	0.2120	0.0743	−0.2172	−0.2380	−0.2558	−0.0478	−0.1590	−0.1014	−0.2333	0.3250	0.2570	0.2322
Component 5	0.0313	0.0669	0.0487	0.2555	0.0235	−0.1512	0.1147	0.3042	−0.0678	0.1503	0.1816	0.2094	0.1634	0.2468	0.0070	−0.2746	0.0330	0.0113	−0.2451

The 5 principal components contributed to 94.36% of the total variance.

The scores are for the conversion between 5 principal components and 38 ingredient variables.

**Table 8:** Relative peak areas of 38 identified ingredients in the eleven KYQG samples.

	**P1**	**P2**	**P3**	**P4**	**P5**	**P6**	**P7**	**P8**	**P9**	**P10**	**P11**	**P12**	**P13**	**P14**	**P15**	**P16**	**P17**	**P18**	**P19**
S1	0.0083	0.2467	0.0502	0.0287	0.0390	0.0193	0.9459	0.0615	1.1893	0.0967	0.1904	0.0000	1.0254	5.7467	1.2295	0.0492	0.1995	0.4112	0.3902
S2	0.0086	0.1784	0.0525	0.0236	0.1198	0.0164	0.6218	0.0497	0.8337	0.0837	0.1763	0.0137	0.7539	5.0650	0.9823	0.0460	0.1803	0.4002	0.3564
S3	0.0050	0.2052	0.0232	0.0187	0.0232	0.0117	0.5363	0.0378	0.7682	0.0578	0.1174	0.0100	0.7289	4.7275	0.9818	0.0348	0.1492	0.3405	0.3161
S4	0.0066	0.1673	0.0351	0.0222	0.0589	0.0131	0.5501	0.0484	0.4935	0.0879	0.1550	0.0117	0.6923	4.9273	0.9296	0.0365	0.1630	0.3623	0.3399
S5	0.0076	0.2456	0.0322	0.0167	0.0246	0.0170	0.5240	0.0342	0.7331	0.0679	0.1475	0.0091	0.6592	4.1273	0.8103	0.0375	0.1136	0.3136	0.2672
S6	0.0032	0.1126	0.0118	0.0108	0.0378	0.0060	0.2897	0.0471	0.7259	0.0820	0.1261	0.0119	1.0891	6.9542	1.4232	0.0383	0.1602	0.4319	0.4384
S7	0.0052	0.1213	0.0263	0.0136	0.1624	0.0089	0.2815	0.0469	0.6969	0.0760	0.1285	0.0189	0.8278	4.9017	1.0951	0.0430	0.1190	0.3424	0.3175
S8	0.0079	0.2525	0.0289	0.0115	0.0451	0.0130	0.3428	0.0362	0.6906	0.0817	0.1521	0.0242	0.6589	4.0349	0.8430	0.0410	0.0869	0.2855	0.2454
S9	0.0000	0.0040	0.0058	0.0027	0.0045	0.0011	0.0097	0.0363	0.5569	0.0045	0.0037	0.0128	0.3114	2.8818	0.5021	0.0048	0.0248	0.0893	0.1738
S10	0.0001	0.0060	0.0042	0.0013	0.0041	0.0019	0.0147	0.0287	0.5091	0.0042	0.1904	0.0000	1.0254	5.7467	1.2295	0.0492	0.1995	0.4112	0.3902
S11	0.0101	0.2246	0.0271	0.0129	0.1243	0.0189	0.3085	0.0494	0.6390	0.0907	0.1763	0.0137	0.7539	5.0650	0.9823	0.0460	0.1803	0.4002	0.3564
	**P20**	**P21**	**P22**	**P23**	**P24**	**P25**	**P26**	**P27**	**P28**	**P29**	**P30**	**P31**	**P32**	**P33**	**P34**	**P35**	**P36**	**P37**	**P38**
S1	1.3639	1.2320	2.8844	0.0000	1.7443	0.0474	0.0233	0.0000	0.0000	0.0371	0.0194	0.0469	0.8648	1.6664	1.6664	0.4191	0.0213	0.0187	0.0304
S2	1.1094	1.0761	2.5344	0.0167	1.3082	0.1186	0.0391	0.0908	0.0090	0.0344	0.0194	0.0449	0.6080	1.4686	1.4339	0.8219	0.0053	0.0134	0.0078
S3	1.1265	0.9833	2.3728	0.0296	1.2500	0.0101	0.0006	0.1319	0.0146	0.0363	0.0201	0.0499	0.7493	1.5654	1.1853	0.2669	0.0226	0.0273	0.0370
S4	1.0150	1.0787	2.3476	0.0503	1.2951	0.0986	0.0339	0.2230	0.0199	0.0340	0.0199	0.0489	0.7201	1.6315	1.2060	0.6666	0.0108	0.0112	0.0127
S5	0.9453	0.8553	2.0386	0.0418	0.7775	0.0000	0.0000	0.2224	0.0248	0.0292	0.0186	0.0441	0.4329	1.0328	0.8605	0.0000	0.0315	0.0156	0.0279
S6	1.4563	1.2943	2.9924	0.0510	1.2646	0.0589	0.0066	0.2955	0.0341	0.0174	0.0111	0.0294	0.6245	0.8991	1.2477	0.7304	0.0258	0.0156	0.0158
S7	1.2508	0.9442	2.4701	0.0481	0.7081	0.1154	0.1234	0.3085	0.0302	0.0322	0.0207	0.0456	0.4881	1.2657	0.9057	1.2909	0.0000	0.0000	0.0000
S8	1.0078	0.8650	2.0646	0.0795	0.4711	0.0504	0.1005	0.3660	0.0430	0.0266	0.0177	0.0431	0.3274	0.8295	0.4560	0.2247	0.0310	0.0550	0.0196
S9	0.5557	0.5582	1.4838	0.0510	0.2562	0.0215	0.0578	0.2065	0.0634	0.0197	0.0143	0.0377	0.0987	0.2975	0.4634	0.8944	0.0184	0.0361	0.0178
S10	1.3639	0.4979	1.2969	0.0536	0.1293	0.0143	0.0259	0.2469	0.0639	0.0172	0.0132	0.0383	0.0603	0.1763	0.2004	0.2248	0.0479	0.0324	0.0501
S11	1.1094	0.6736	1.6604	0.0846	0.0000	0.1053	0.0627	0.3806	0.0601	0.0210	0.0173	0.0407	0.0000	0.0000	0.0000	1.0439	0.0193	0.0385	0.0387

S1-S11 represented the eleven KYQG samples.

P1-P38 represented 38 identified ingredients in KYQG and were regarded as the independent ingredient variables.

**Table 9:** Dimensionless data of the 38 relative peak areas in the eleven KYQG samples.

	**P1**	**P2**	**P3**	**P4**	**P5**	**P6**	**P7**	**P8**	**P9**	**P10**	**P11**	**P12**	**P13**	**P14**	**P15**	**P16**	**P17**	**P18**	**P19**
S1	1.4622	1.5383	1.8585	1.9385	0.6657	1.6695	2.3513	1.4201	1.6695	1.4514	1.5582	0.0000	1.4559	1.2845	1.3398	1.4604	1.8137	1.3947	1.3507
S2	1.5144	1.1124	1.9402	1.5950	2.0476	1.4198	1.5457	1.1486	1.1703	1.2555	1.4425	1.0117	1.0703	1.1322	1.0704	1.3633	1.6388	1.3573	1.2335
S3	0.8742	1.2796	0.8594	1.2632	0.3968	1.0122	1.3332	0.8730	1.0783	0.8669	0.9605	0.7359	1.0349	1.0567	1.0699	1.0308	1.3564	1.1547	1.0939
S4	1.1642	1.0434	1.2967	1.5031	1.0070	1.1326	1.3675	1.1195	0.6927	1.3185	1.2682	0.8637	0.9829	1.1014	1.0130	1.0828	1.4818	1.2288	1.1763
S5	1.3425	1.5313	1.1915	1.1265	0.4202	1.4700	1.3025	0.7895	1.0291	1.0191	1.2072	0.6726	0.9359	0.9226	0.8830	1.1124	1.0324	1.0637	0.9248
S6	0.5610	0.7018	0.4383	0.7311	0.6464	0.5215	0.7202	1.0877	1.0189	1.2303	1.0321	0.8808	1.5462	1.5544	1.5509	1.1356	1.4565	1.4647	1.5175
S7	0.9056	0.7563	0.9744	0.9179	2.7759	0.7667	0.6998	1.0832	0.9783	1.1408	1.0518	1.3969	1.1753	1.0956	1.1934	1.2750	1.0820	1.1611	1.0988
S8	1.3803	1.5740	1.0674	0.7787	0.7701	1.1242	0.8521	0.8365	0.9694	1.2253	1.2444	1.7915	0.9355	0.9019	0.9187	1.2149	0.7899	0.9684	0.8494
S9	0.0052	0.0251	0.2148	0.1822	0.0764	0.0921	0.0240	0.8379	0.7817	0.0682	0.0299	0.9494	0.4422	0.6441	0.5472	0.1414	0.2252	0.3028	0.6014
S10	0.0092	0.0374	0.1560	0.0893	0.0696	0.1606	0.0366	0.6631	0.7146	0.0629	0.0427	0.3502	0.5145	0.5798	0.5839	0.1038	0.1233	0.2462	0.5025
S11	1.7811	1.4004	1.0028	0.8743	2.1243	1.6308	0.7670	1.1409	0.8971	1.3612	1.1625	2.3473	0.9064	0.7268	0.8298	1.0797	0.0000	0.6576	0.6512
	**P20**	**P21**	**P22**	**P23**	**P24**	**P25**	**P26**	**P27**	**P28**	**P29**	**P30**	**P31**	**P32**	**P33**	**P34**	**P35**	**P36**	**P37**	**P38**
S1	1.3225	1.3473	1.3140	0.0000	2.0845	0.8143	0.5403	0.0000	0.0000	1.3379	1.1120	1.0993	1.9124	1.6921	1.9044	0.7002	1.0008	0.7806	1.2959
S2	1.0757	1.1769	1.1546	0.3633	1.5634	2.0370	0.9088	0.4039	0.2724	1.2386	1.1129	1.0511	1.3445	1.4913	1.6387	1.3732	0.2472	0.5571	0.3327
S3	1.0923	1.0753	1.0810	0.6426	1.4939	0.1730	0.0142	0.5869	0.4422	1.3088	1.1534	1.1689	1.6570	1.5896	1.3546	0.4460	1.0630	1.1377	1.5776
S4	0.9842	1.1796	1.0695	1.0932	1.5478	1.6941	0.7867	0.9923	0.6036	1.2272	1.1425	1.1457	1.5926	1.6567	1.3783	1.1138	0.5101	0.4656	0.5431
S5	0.9166	0.9354	0.9287	0.9086	0.9292	0.0000	0.0000	0.9898	0.7513	1.0531	1.0675	1.0330	0.9573	1.0488	0.9834	0.0000	1.4793	0.6527	1.1918
S6	1.4121	1.4155	1.3632	1.1078	1.5113	1.0113	0.1528	1.3149	1.0345	0.6263	0.6347	0.6877	1.3811	0.9130	1.4259	1.2203	1.2127	0.6514	0.6736
S7	1.2128	1.0326	1.1253	1.0446	0.8462	1.9813	2.8657	1.3727	0.9155	1.1596	1.1860	1.0693	1.0793	1.2853	1.0350	2.1568	0.0000	0.0000	0.0000
S8	0.9772	0.9460	0.9406	1.7274	0.5630	0.8648	2.3324	1.6284	1.3018	0.9601	1.0175	1.0107	0.7240	0.8423	0.5211	0.3755	1.4571	2.2937	0.8344
S9	0.5388	0.6104	0.6760	1.1082	0.3062	0.3698	1.3430	0.9190	1.9226	0.7092	0.8224	0.8837	0.2184	0.3021	0.5296	1.4944	0.8665	1.5048	0.7610
S10	0.5779	0.5445	0.5908	1.1647	0.1546	0.2464	0.6012	1.0985	1.9364	0.6215	0.7595	0.8969	0.1334	0.1790	0.2290	0.3756	2.2544	1.3498	2.1375
S11	0.8899	0.7366	0.7564	1.8396	0.0000	1.8081	1.4549	1.6936	1.8197	0.7576	0.9916	0.9538	0.0000	0.0000	0.0000	1.7442	0.9090	1.6067	1.6525

S1-S11 represented the eleven KYQG samples.

P1-P38 represent 38 identified ingredients in KYQG and were regarded as the independent ingredient variables. P1-P38 were processed to be dimensionless by the equalization method for the calculation.

**Table 10:** Switching of the effect parameters from negative to positive.

	**IL-1β**	**TNF-α**	**IL-8**	**IL-6**
S1	0.0528	0.8706	0.0035	0.4214
S2	0.0554	0.6100	0.0038	0.3959
S3	0.0632	0.6317	0.0038	0.3484
S4	0.0679	0.8206	0.0029	0.3959
S5	0.0627	0.6671	0.0040	0.3787
S6	0.0793	0.8923	0.0042	0.3959
S7	0.0573	0.5171	0.0040	0.3580
S8	0.0763	0.5664	0.0029	0.4665
S9	0.0703	0.5664	0.0035	0.3900
S10	0.0697	0.5286	0.0039	0.3843
S11	0.0735	0.7139	0.0036	0.4749

S1-S11 represented the eleven KYQG samples.

Negative effect values (the smaller the better) were converted to positive values by taking the reciprocal.

**Table 11:** Dimensionless data of the effect parameters after switching from negative to positive.

	**IL-1β**	**TNF-α**	**IL-8**	**IL-6**
S1	0.7981	1.2968	0.9497	1.0511
S2	0.8364	0.9087	1.0364	0.9875
S3	0.9539	0.9409	1.0540	0.8692
S4	1.0260	1.2223	0.8062	0.9875
S5	0.9465	0.9937	1.0997	0.9446
S6	1.1969	1.3292	1.1611	0.9875
S7	0.8660	0.7702	1.0890	0.8929
S8	1.1518	0.8437	0.8048	1.1636
S9	1.0617	0.8437	0.9544	0.9728
S10	1.0526	0.7874	1.0612	0.9585
S11	1.1099	1.0633	0.9834	1.1847

S1-S11 represented the eleven KYQG samples.

Effect values of switched parameters were processed to be dimensionless by an equalization method for the calculation.

## References

[b1] Jiang W. Y. Therapeutic wisdom in traditional Chinese medicine: a perspective from modern science. Trends Pharmacol. Sci. 26, 558–563 (2005). 16185775 10.1016/j.tips.2005.09.006

[b2] Xue R. *et al.* TCMID: traditional Chinese medicine integrative database for herb molecular mechanism analysis. Nucleic Acids Res. 41, D1089–D1095 (2013). 23203875 10.1093/nar/gks1100 PMC3531123

[b3] Wang X. *et al.* Ultra high performance liquid chromatography with tandem mass spectrometry method for the determination of tetrandrine and fangchinoline in rat plasma after oral administration of Fangji Huangqi Tang and Stephania tetrandra S. Moore extracts. J. Sep. Sci. 38, 1286–1293 (2015). 25645647 10.1002/jssc.201401384

[b4] Lau K. M. *et al.* Synergistic interaction between Astragali Radix and Rehmanniae Radix in a Chinese herbal formula to promote diabetic wound healing. J. Ethnopharmacol. 141, 250–256 (2012). 22366433 10.1016/j.jep.2012.02.025

[b5] Wang M. *et al.* Metabolomics in the context of systems biology: bridging traditional Chinese medicine and molecular pharmacology. Phytother. Res. 19, 173–182 (2005). 15934013 10.1002/ptr.1624

[b6] Tao W. *et al.* Network pharmacology-based prediction of the active ingredients and potential targets of Chinese herbal Radix Curcumae formula for application to cardiovascular disease. J. Ethnopharmacology 145, 1–10 (2013). 23142198 10.1016/j.jep.2012.09.051

[b7] Kitts D. D. & Hu C. Efficacy and safety of ginseng. Public Health Nutr. 3, 473–485 (2000). 11276295 10.1017/s1368980000000550

[b8] Li R. *et al.* Creation of fingerprint efficacy relevancy for traditional Chinese medicine. J. Educ. Chin. Med. 2, 62 (2002).

[b9] Liu H. *et al.* Core bioactive components promoting blood circulation in the traditional Chinese medicine compound Xueshuantong Capsule (CXC) based on the relevance analysis between chemical HPLC fingerprint and *in vivo* biological effects. PloS One 9, e112675 (2014). 25396725 10.1371/journal.pone.0112675 PMC4232446

[b10] Wang J. *et al.* Bioactive components on immuno-enhancement effects in the traditional Chinese medicine Shenqi Fuzheng Injection based on relevance analysis between chemical HPLC fingerprints and *in vivo* biological effects. J. Ethnopharmacology 155, 405–415 (2014). 24950446 10.1016/j.jep.2014.05.038

[b11] Li P., Li X., Chen J. W., Wu M. X. & Chen C. Y. Spectrum-effect relationship of active component from Taohong Siwutang in dysmenorrheal model mice. China J. Exper. Trad. Med. Form. 16, 144–149 (2010).

[b12] Qin K. M. *et al.* Application of spectrum-effect relationship in Chinese medicine research and related thinking. China J. Chin. Mat. Med. 38, 26–31(2013). 23596870

[b13] Yan S. K. *et al.* Chemometrics-based approach to modeling quantitative composition-activity relationships for Radix Tinosporae. Interdiscip. Sci. Comput. Life Sci. 2, 221–227 (2010). 10.1007/s12539-010-0026-9 20658334

[b14] Li Z. S. & Zhang X. N. The pharmacodynamic study of Kouyanqing. Trad. Chin. Drug Res. Clin. Pharm. 10, 216–217 (1999).

[b15] Gu N., Ni L. & Fan Y. Influence of Kouyanqing Granules on serum immunoglobulin of patients with recurrent aphthous ulceration. China Med. Her. 21, 018 (2010).

[b16] Liang Y. Z., Fang K. T. & Xu Q. S. Uniform design and its applications in chemistry and chemical engineering. Chemom. Intell. Lab. Syst. 58, 43–57 (2001).

[b17] Hao J. F., Guo J. Z., Xie F. W., Xia Q. L. & Xie J. P. Influences of pyrolysis condidtions on the major pyrolytic products of asparagine. J. Instru. Anal. 32, 519–528 (2013).

[b18] Soga T., Kakazu Y., Robert M., Tomita M. & Nishioka T. Qualitative and quantitative analysis of amino acids by capillary electrophoresis-electrospray ionization-tandem mass spectrometry. Electrophoresis 25, 1964–1972 (2004). 15237395 10.1002/elps.200305791

[b19] Xu N. *et al.* Direct detection of amino acids using extractive electrospray ionization tandem mass spectrometry. Chin. J. Anal. Chem. 41, 523–528 (2013).

[b20] Qu j. *et al.* Rapid determination of underivatized pyroglutamic acid,glutamic acid, glutamine and other relevant amino acids in fermentation media by LC-MS-MS. Analyst 127, 66–69 (2002). 11827398 10.1039/b108422b

[b21] Fang N., Yu S. & Prior R. L. LC/MS/MS characterization of phenolic constituents in dried plums. J. Agric. Food Chem. 50, 3579–3585 (2002). 10.1021/jf0201327 12033832

[b22] Iwai K., Kishimoto N., Kakino Y., Mochida K. & Fujita T. *In Vitro* Antioxidative effects and tyrosinase inhibitory activities of seven hydroxycinnamoyl derivatives in green coffee beans. J. Agric. Food Chem. 52, 4893–4898 (2004). 15264931 10.1021/jf040048m

[b23] Zeper A. *et al.* Analysis of sterroidal saponins in crude extract from asparagus cochinchinensis by mass spectrometry. J. Instru. Anal. 24, 52–53 (2005).

[b24] Chen J., Xu X. F., Chai X. Y. & Li P. Chemical Constituents in the Buds of Lonicera macranthoides. Chin. J. Nat. Med. 4, 347–351 (2006).

[b25] Lin Y. J. *et al.* Changes of chemical ingredients before and after compatibility of *Aconiti Lateralis Radix Praeparata* and *Glycyrrhizae Radix* et *Rhizoma Praeparata* cum *Melle* analyzed by HPLC-Q-TOF/MS fingerprint technology. *Chin. Trad. Herbal* Drugs 45, 1556–1560 (2014).

[b26] Jeong S. H. *et al.* Up-regulation of TNF-alpha secretion by cigarette smoke is mediated by Egr-1 in HaCaT human keratinocytes. Exp. Dermatol. 19, e206–e212 (2010). 20653771 10.1111/j.1600-0625.2009.01050.x

[b27] Rohrer J., Wuertz B. R. & Ondrey F. Cigarette smoke condensate induces nuclear factor kappa-b activity and proangiogenic growth factors in aerodigestive cells. The Laryngoscope 120, 1609–1613 (2010). 20564670 10.1002/lary.20972

[b28] Semlali A., Witoled C., Alanazi M. & Rouabhia M. Whole cigarette smoke increased the expression of TLRs, HBDs, and proinflammory cytokines by human gingival epithelial cells through different signaling pathways. PloS One 7, e52614 (2012). 23300722 10.1371/journal.pone.0052614 PMC3532503

[b29] Dinarello C. A. Proinflammatory cytokines. Chest J. 118, 503–508 (2000). 10.1378/chest.118.2.503 10936147

[b30] Turner N. A. *et al.* Mechanism of TNFα-induced IL-1α, IL-1β and IL-6 expression in human cardiac fibroblasts: effects of statins and thiazolidinediones. Cardiovasc. Res. 76, 81–90 (2007). 17612514 10.1016/j.cardiores.2007.06.003

[b31] Sun A., Wang J. T., Chia J. S. & Chiang C. P. Serum interleukin-8 level is a more sensitive marker than serum interleukin-6 level in monitoring the disease activity of oral lichen planus. Brit. J. Dermatol. 152, 1187–1192 (2005). 15948980 10.1111/j.1365-2133.2005.06497.x

[b32] Wang X., Feuerstein G. Z., Gu J. L., Lysko P. G. & Yue T. L. Interleukin-1β induces expression of adhesion molecules in human vascular smooth muscle cells and enhances adhesion of leukocytes to smooth muscle cells. Atherosclerosis 115, 89–98 (1995). 7545398 10.1016/0021-9150(94)05503-b

[b33] Rhodus N. L. *et al.* Proinflammatory cytokine levels in saliva before and after treatment of (erosive) oral lichen planus with dexamethasone. Oral Dis. 12, 112–116 (2006). 16476030 10.1111/j.1601-0825.2005.01165.x

[b34] Xavier G. M., Sá A. R. D., Guimarães A. L. S., Silva T. A. D. & Gomez R. S. Investigation of functional gene polymorphisms interleukin-1β, interleukin-6, interleukin-10 and tumor necrosis factor in individuals with oral lichen planus. J. Oral Pathol. Med. 36, 476–481 (2007). 17686006 10.1111/j.1600-0714.2007.00560.x

[b35] Gupta P., Ashok L. & Naik S. R. Assessment of serum interleukin-8 as a sensitive serological marker in monitoring the therapeutic effect of levamisole in recurrent aphthous ulcers: A randomized control study. Indian J. Dent. Res. 25, 284 (2014). 25098981 10.4103/0970-9290.138293

[b36] Scully C. & Porter S. Oral mucosal disease: recurrent aphthous stomatitis. Brit. J. Oral Max. Surg. 46, 198–206 (2008). 10.1016/j.bjoms.2007.07.201 17850936

[b37] Tobita T., Izumi K. & Feinberg S. E. Development of an *in vitro* model for radiation-induced effects on oral keratinocytes. Int. J Oral Max. Surg. 39, 364–370 (2010). 10.1016/j.ijom.2009.12.020 PMC2859991 20080035

[b38] Wei G. W. Grey relational analysis method for 2-tuple linguistic multiple attribute group decision making with incomplete weight information. Expert Syst. Appl. 38, 4824–4828 (2011).

[b39] Kuo Y., Yang T. & Huang G. W. The use of grey relational analysis in solving multiple attribute decision-making problems. Comput. Ind. Eng. 55, 80–93 (2008).

[b40] Yu J. P., Huang X. M. & Xia X. Y. Image Data Mining Technology of Multimedia in *Future Computing, Communication, Control and Managem*ent (ed. Zhang Y.) 379–385 (Springer, 2012).

[b41] Kim H. G. & Oh M. S. Natural products as potential anticonvulsants: caffeoylquinic acids. Arch. Pharm. Res. 35, 389–392 (2012). 22477183 10.1007/s12272-012-0300-y

[b42] Zhao Z., Shin H. S., Satsu H., Totsuka M. & Shimizu M. 5-Caffeoylquinic acid and caffeic acid down-regulate the oxidative stress-and TNF-α-induced secretion of interleukin-8 from Caco-2 cells. J. Agr. Food Chem. 56, 3863–3868 (2008). 18444659 10.1021/jf073168d

[b43] Hwang S. J., Kim Y. W., Park Y., Lee H. J. & Kim K. W. Anti-inflammatory effects of chlorogenic acid in lipopolysaccharide-stimulated RAW 264.7 cells. Inflamm. Res. 63, 81–90 (2014). 24127072 10.1007/s00011-013-0674-4

[b44] Li Y. *et al.* Screening and analyzing the potential bioactive components from reduning injection, using macrophage cell extraction and ultra-high performance liquid chromatography coupled with mass spectrometry. Am. J. Chinese Med. 41, 221–229 (2013). 10.1142/S0192415X1350016X 23336518

[b45] Hong S., Joo T. & Jhoo J. W. Antioxidant and anti-inflammatory activities of 3, 5-dicaffeoylquinic acid isolated from Ligularia fischeri leaves. Food Sci. Biotechnol. 24, 257–263 (2015).

[b46] Morikawa K. *et al.* Inhibitory effect of quercetin on carrageenan-induced inflammation in rats. Life Sci. 74, 709–721 (2003). 14654164 10.1016/j.lfs.2003.06.036

[b47] Xagorari A. *et al.* Luteolin inhibits an endotoxin-stimulated phosphorylation cascade and proinflammatory cytokine production in macrophages. J. Pharmacol. Exp. Ther. 296, 181–187 (2001). 11123379

[b48] Inaba K., Murata K., Naruto S. & Matsuda H. Inhibitory effects of devil’s claw (secondary root of Harpagophytum procumbens) extract and harpagoside on cytokine production in mouse macrophages. J. Nat. Med. 64, 219–222 (2010). 20177800 10.1007/s11418-010-0395-8

[b49] Háznagy-Radnai E. *et al.* Antiinflammatory activities of Hungarian Stachys species and their Iridoids. Phytother. Res. 26, 505–509 (2012). 21887806 10.1002/ptr.3582

[b50] Díaz A. M. *et al.* Phenylpropanoid glycosides from Scrophularia scorodonia: *in vitro* anti-inflammatory activity. Life Sci. 74, 2515-2526 (2004). 15010262 10.1016/j.lfs.2003.10.008

[b51] Sun Q. *et al.* Ruscogenin inhibits lipopolysaccharide-induced acute lung injury in mice: Involvement of tissue factor, inducible NO synthase and nuclear factor (NF)-κB. Inter. Immunopharm. 12, 88–93 (2012). 10.1016/j.intimp.2011.10.018 22079591

[b52] Lu H. J. *et al.* Ruscogenin ameliorates diabetic nephropathy by its anti-inflammatory and anti-fibrotic effects in streptozotocin-induced diabetic rat. BMC Comple. Alter. Med. 14, 110 (2014). 10.1186/1472-6882-14-110 PMC3986976 24666993

[b53] Kim D. S. *et al.* The therapeutic effect of chelidonic acid on ulcerative colitis. Biol. Pharm. Bull. 35, 666–671 (2012). 22687399 10.1248/bpb.35.666

[b54] Han D., Kim H. Y., Lee H. J., Shim I. & Hahm D. H. Wound healing activity of gamma-aminobutyric Acid (GABA) in rats. J. Microbiol. Biotechnol. 17, 1661–1669 (2007). 18156782

[b55] Zhang X., Tong Z., Zhou, Y. J. & Xu X. Studies on medicinal ingredients and pharmacological effects of Lonicera. Chin. Pharmacol. Bull. 30, 1049–1054 (2014).

[b56] Roach J. D. Jr, Aguinaldo G. T., Jonnalagadda, K., Hughes F. M. Jr & Spangelo B. L. γ-Aminobutyric Acid Inhibits Synergistic Interleukin-6 Release sbut not Transcriptional Activation in Astrocytoma Cells. Neuroimmunomodulation 15, 117 (2008). 18679050 10.1159/000148194 PMC2859952

[b57] Meyers B. E., Moonka D. K. & Davis R. H. The effect of selected amino acids on gelatin-induced inflammation in adult male mice. Inflammation 3, 225–233 (1979). 478594 10.1007/BF00914179

[b58] Tang G. J. *et al.* Novel role of AMP-activated protein kinase signaling in cigarette smoke induction of IL-8 in human lung epithelial cells and lung inflammation in mice. Free Rad. Bio. Med. 50, 1492–1502 (2011). 21376115 10.1016/j.freeradbiomed.2011.02.030

[b59] Yang S. R. *et al.* Cigarette smoke induces proinflammatory cytokine release by activation of NF-κB and posttranslational modifications of histone deacetylase in macrophages. Am. J. Physiol-Lung C. 291, L46–L57 (2006). 10.1152/ajplung.00241.2005 16473865

[b60] Nakamura Y. *et al.* Cigarette smoke inhibits lung fibroblast proliferation and chemotaxis. Am. J. Respir. Crit. Care Med. 151, 1497–1503 (1995). 10.1164/ajrccm.151.5.7735606 7735606

[b61] Hamzaçebi C., Akay D. & Kutay F. Comparison of direct and iterative artificial neural network forecast approaches in multi-periodic time series forecasting. Expert Syst. Appl. 36, 3839–3844 (2009).

[b62] Dai J. J., Lieu L. & Rocke D. Dimension reduction for classification with gene expression microarray data. Stat. Appl. Genet. Mol. Biol. 5, 1–21 (2006). 10.2202/1544-6115.1147 16646870

[b63] Wang H., Liu Q. & Tu Y. Interpretation of partial least-squares regression models with VARIMAX rotation. Comput. Stat. Data An. 48, 207–219 (2005).

