# Supplementary material for: Discovery of Anti-inflammatory Ingredients in Chinese Herbal Formula Kouyanqing Granule based on Relevance Analysis between Chemical Characters and Biological Effects
Source: Sci Rep. 2015 Dec 10;5:18080. doi: 10.1038/srep18080 (PMC4674803; doi:10.1038/srep18080)
Supplement: Supplementary Information [file srep18080-s1.pdf]

Manuscript ID: SREP-15-18150A

Title: Discovery of Anti-inflammatory Ingredients in Chinese Herbal Formula Kouyanqing Granule based on Relevance Analysis between Chemical Characters and Biological Effects

Authors: Hong Liu[1,†], Yan-fang Zheng[1,†], Chu-yuan Li[2], Yu-ying Zheng[1], De-qin Wang[2], Zhong Wu[1], Lin Huang[2], Yong-gang Wang[1], Pei-bo Li[1], Wei Peng[1], Wei-wei Su[1,*]

[†] **Two authors contributed equally to this work.**

[1] Guangzhou Quality R&D Center of Traditional Chinese Medicine, Guangdong Key Laboratory of Plant Resources, School of Life Sciences, Sun Yat-sen University, Guangzhou, P.R. China

[2] Hutchison Whampoa Guangzhou Baiyunshan Chinese Medicine Co., Ltd., Guangzhou, P.R. China

Running title: Bioactive Ingredients for Anti-inflammatory Effects of Kouyanqing Granule

Correspondence: [*]Wei-wei Su, Guangzhou Quality R&D Center of Traditional Chinese Medicine, Guangdong Key Laboratory of Plant Resources, School of Life Sciences, Sun Yat-sen University, 135 Xingangxi Road, Guangzhou 510275, PR China. Tel: +86 20 84110808, Fax: +86 20 84112398, E-mail: lsssww@126.com

Supplementary Information-Video Legends

KYQG ingredient-effect relevance bubble chart S1: a dynamic bubble chart based on the computed results (Table 6) to illustrate a clear picture of the anti-inflammatory effects of the 38 identified ingredients in KYQG. Y-axis: Partial least-squares regression coefficient; X-axis: Grey relational degree; Span: Effect parameters (IL-1β, IL-6, IL-8, TNF-α); Bubble ID: 38 Identified ingredients; Bubble Color: 38 Identified ingredients.

KYQG herb-effect relevance bubble chart S2: a dynamic bubble chart based on the computed results (Table 6) to illustrate a clear picture of the anti-inflammatory effects of the 5 herbs in KYQG. Y-axis: Partial least-squares regression coefficient; X-axis: Grey relational degree; Span: Effect parameters (IL-1β, IL-6, IL-8, TNF-α); Bubble ID: 38 Identified ingredients; Bubble Color: 5 herbs.
